# Associations of Accelerated DNA Methylation Aging Algorithms with Chronic Liver Disease and All-Cause Mortality

**DOI:** 10.3390/biomedicines14081792

**Published:** 2026-08-09

**Authors:** Xinyi Zhang, Zhenduo Chen, Zike Cheng, Hui Zhang, Yaqian Xu, Chongyu Ding, Tongyan An, Yulu Gong, Darong Hao, Jinjie Xu, Shuaiyin Chen

**Affiliations:** 1 College of Public Health, Zhengzhou University, Zhengzhou 450001, China; zhangxinyi_1216@163.com (X.Z.); antongyan0811@163.com (T.A.); 2 Yantai Center for Disease Control and Prevention, Yantai 264003, China; zhenduochen@163.com; 3 Binzhou Center for Disease Control and Prevention, Binzhou 256600, China; jackcheng18@163.com; 4 School of Global Health, Chinese Center for Tropical Diseases Research, Shanghai 200025, China; zhanghui2939@163.com (H.Z.); xuyq1113@sjtu.edu.cn (Y.X.); cyding@sjtu.edu.cn (C.D.); catherine_1417@163.com (Y.G.); haodarong@163.com (D.H.); 5 Hainan International Medical Center, Shanghai Jiao Tong University School of Medicine, Qionghai 571434, China; 6 School of Public Health, Shanghai Jiao Tong University School of Medicine, Shanghai 200025, China

**Keywords:** chronic liver disease, DNA methylation, epigenetics, biological ageing, all-cause mortality

## Abstract

**Background:** Chronic liver disease (CLD) represents a substantial global health challenge, with significant morbidity and mortality worldwide. Biological aging may be involved in CLD-related outcomes, but the associations of diverse DNA methylation (DNAm) aging algorithms with CLD phenotypes and long-term mortality remain incompletely characterized. **Methods:** Using a US nationally representative cohort, we analyzed 12 DNAm aging algorithms in 2522 adults aged ≥50. CLD was classified into viral, alcohol-related, metabolic syndrome (MetS)-related liver disease, or uncharacterized groups. Advanced fibrosis was defined by AST-to-platelet ratio index ≥0.7. Associations of algorithms with CLD and all-cause mortality (followed through 2019) were assessed using multivariable-adjusted regression and logistic regression models and Cox models, accounting for complex sampling. **Results:** Among participants with CLD, DNAm aging algorithms showed only nominal associations with APRI-defined advanced fibrosis. GrimAgeMortAcc, GrimAge2MortAcc, HannumAgeAcc, PhenoAgeAcc, DunedinPoAm, and HorvathTelo were significantly associated with all-cause mortality. GrimAge-based measures and PhenoAgeAcc showed the strongest associations with all-cause mortality across CLD-related phenotypes (HRs ranged from 1.31 to 1.82). HorvathTelo was inversely associated with mortality risk (HR = 0.71, 95% CI: 0.62–0.82). **Conclusions:** DNAm aging algorithms, particularly GrimAge-based measures, are strongly associated with long-term all-cause mortality among individuals with CLD-related phenotypes. Although associations with fibrosis-related outcomes varied according to fibrosis definitions, DNAm aging algorithms may provide additional biological information for mortality risk stratification among individuals with CLD-related phenotypes.

## 1. Introduction

Chronic liver disease (CLD) is a leading global cause of morbidity and mortality, resulting in over two million deaths annually and ranking as the eleventh leading cause of death both in the United States and globally [1]. CLD arises from diverse etiologies such as viral hepatitis, alcoholic liver disease, and metabolic dysfunction-associated steatotic liver disease (MASLD) [2]. Notably, regardless of the initial cause, these conditions may ultimately progress to liver fibrosis, cirrhosis, and hepatocellular carcinoma, driven largely by chronic inflammation, hepatocyte senescence, and extracellular matrix deposition [1,3]. Among these drivers, cellular senescence has been recognized as a central mechanism underlying CLD initiation and progression [4].

The ongoing accumulation of such cellular senescence ultimately manifests at a systemic level as an acceleration of biological age [5]. Biological age is a multidimensional indicator of functional integrity, distinct from chronological age, and can be quantified through epigenetic markers such as DNA methylation (DNAm) patterns [6]. Persistent cellular damage and stress accelerate this biological aging process, which in turn activates profound pro-aging pathways [7], including the accumulation of senescent cells, mitochondrial dysfunction, and nutrient-sensing dysregulation. These changes which collectively drive inflammation, impair regeneration, and exacerbate fibrosis [8].

Recent advances in epigenetic profiling, particularly the development of DNAm aging algorithms, have made it possible to accurately quantify biological aging [9]. Built upon these advances, these DNAm aging algorithms have evolved into several generations based on their design objectives. First-generation DNAm aging algorithms (e.g., Horvath, Hannum) primarily estimate chronological age [10,11], second-generation models (e.g., PhenoAge, GrimAge, GrimAge2Mort) incorporate morbidity and mortality risk factors to more directly reflect health status [12,13,14], and emerging third-generation (e.g., DunedinPACE) aims to capture the pace of physiological aging [15]. The discrepancy between DNAm age and chronological age, termed age acceleration (AA), has demonstrated robust associations with age-related diseases, including CLD [16,17]. For instance, HorvathAge acceleration has been associated with MASLD, and GrimAge acceleration strongly predicts both hepatic steatosis and mortality risk, often outperforming earlier DNAm aging algorithms [18].

Despite these advances, there remains a lack of systematic comparative evaluation of different DNAm aging algorithms across the spectrum of CLD. In particular, it remains unclear whether different DNAm aging algorithms show distinct associations with CLD etiologies, fibrosis severity, and long-term all-cause mortality. Therefore, we aimed to systematically evaluate the associations of twelve DNAm aging algorithms with overall CLD, major CLD etiologies, APRI-defined advanced fibrosis, and all-cause mortality among individuals with CLD-related phenotypes in a nationally representative US population.

## 2. Materials and Methods

### 2.1. Study Population

This study used data from the 1999–2000 and 2001–2002 cycles of the National Health and Nutrition Examination Survey (NHANES), a nationally representative survey of the noninstitutionalized US population using a stratified, multistage probability sampling design. All participants provided written informed consent, and the survey protocols were approved by the National Center for Health Statistics Research Ethics Review Board.

**Figure 1:**
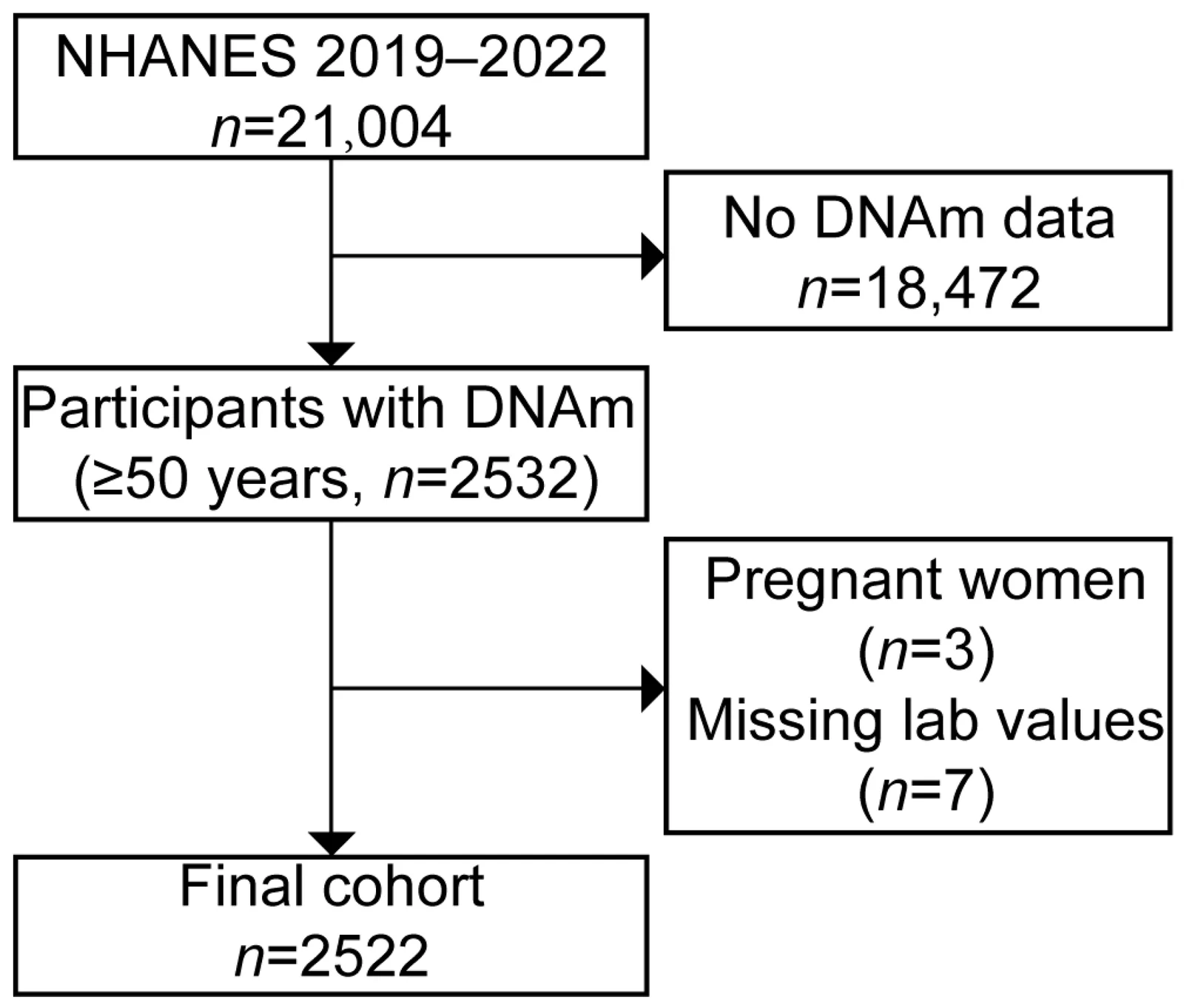
Flowchart of participant selection in the study. Abbreviation: DNAm, DNA methylation; NHANES, the National Health and Nutrition Examination Survey.

Our analyses are restricted to two cycles collected from 1999 to 2002. Of 21,004 eligible participants, 18,472 were excluded due to the lack of DNAm data (available only for adults ≥ 50), leaving a group of 2532 participants. The final analysis cohort (*n* = 2522) excluded pregnant individuals (*n* = 3) which will influence metabolic and epigenetic profiles and any participants who lacked laboratory tests vital for defining CLD (*n* = 7) such as platelet count, or alanine aminotransferase (ALT), and aspartate aminotransferase (AST) levels (Figure 1). The participant selection process is illustrated in Figure 1.

### 2.2. DNAm Algorithms of Aging

According to the DNAm chip data and epigenetic biomarker data documentation for 1999–2002 and previous research [19,20], the analysis focused on participants aged 50 years and above. Blood samples were collected via venipuncture for DNA purification and stored at −80 °C after extraction. DNA was then processed using bisulfite conversion with the Zymo EZ DNA Methylation Kit (Zymo Research, Irvine, CA, USA). DNAm was assessed using the Infinium Methylation EPIC BeadChip kit (EPIC, Illumina, San Diego, CA, USA), which is a high-density DNAm array designed to measure DNAm levels at >850,000 CpG sites across the genome. Measurements were performed in Dr Yongmei Liu’s laboratory at Duke University, following the manufacturer’s instructions. DNAm aging measures were obtained directly from the publicly released NHANES DNAm Epigenetic Biomarker dataset; the present study did not independently preprocess raw IDAT files or CpG-level methylation data. According to the official NHANES documentation, raw EPIC BeadChip intensity data underwent background subtraction and color-channel correction using array control probes. Samples with median signal intensities below 10.5 in both the methylated and unmethylated channels were excluded from epigenetic biomarker production. Missing CpG values required for individual biomarkers were imputed using either the Horvath gold-standard reference means or mean beta values calculated from the NHANES dataset, according to the procedures specified by the developers of each biomarker. Probe-type bias was addressed using beta-mixture quantile normalization. A modified BMIQ procedure based on the Horvath gold-standard reference was used for HorvathAge, HannumAge, SkinBloodAge, PhenoAge, GrimAge, and GrimAge2, whereas the original BMIQ procedure was used for the remaining biomarkers. Detailed preprocessing procedures and source code are provided in the official NHANES 1999–2002 DNA Methylation Array and Epigenetic Biomarkers Data Documentation [20].

Consistent with established DNAm aging research methodology [19,21], we selected 12 DNAm algorithms of aging in the dataset, including HorvathAge [11], HannumAge [10], SkinBloodAge [22], PhenoAge [12], LinAge [23], WeidnerAge [24], VidalBraloAge [25], ZhangAge [26], GrimAgeMort [14], GrimAge2Mort [13], DunedinPoAm [15], and HorvathTelo [27]. Additionally, we calculated the residual values of ten primary DNAm aging algorithms against chronological age to create acceleration biomarkers [19], referred to as HorvathAgeAcc, HannumAgeAcc, SkinBloodAgeAcc, PhenoAgeAcc, LinAgeAcc, WeidnerAgeAcc, VidalBraloAgeAcc, ZhangAgeAcc, GrimAgeMortAcc, GrimAge2MortAcc, which reflect biological aging beyond chronological age.

### 2.3. Definition of CLD

The CLD was defined using laboratory values and questionnaire responses, and subsequently classified into viral, alcohol-associated, metabolic dysfunction (MetS)-related, or uncharacterized categories. Initially, any participant with evidence of active infection with either hepatitis B virus (positive for hepatitis B surface antigen), or hepatitis C virus (HCV; positive for HCV RNA or positive for antibodies to HCV with detectable HCV RNA) was labeled as having viral liver disease (*n* = 35). Next, possible alcohol-associated liver disease (*n* = 316) was defined by elevated aminotransferases (ALT > 19 IU/L for females, >29 IU/L for males) [28] and evidence of either (1) current heavy alcohol use (≥3 drinks per day for females, ≥4 drinks per day for males, or binge drinking [≥4 drinks on same occasion for females, ≥5 drinks on same occasion for males] on 5 or more days per month), (2) current moderate alcohol use (≥2 drinks per day for females, ≥3 drinks per day for males, or binge drinking ≥2 days per month), or (3) a history of daily binge drinking. From the remaining participants, MetS-related liver disease (*n* = 441) was assigned to individuals with elevated ALT or AST (>19 IU for females and >29 IU for males) who also met the criteria for MetS, defined by the presence of at least three of the following: (1) impaired glycemic control (hemoglobin A1c ≥ 5.7%, or fasting serum glucose ≥ 100 mg/dL, or use of diabetes medications), (2) either increased waist circumference (≥88 cm for females, ≥101 cm for males) or elevated body mass index (≥30 Kg/m^2^), (3) low high-density lipoprotein cholesterol (<50 mg/dL in females, <40 mg/dL in males), (4) high triglyceride levels (≥150 mg/dL), or (5) hypertension (systolic blood pressure ≥ 130 mm Hg or diastolic blood pressure ≥ 85 mm Hg). Finally, a further group of uncharacterized liver disease (*n* = 231) consisted of individuals with abnormal aminotransferases (ALT > 25 IU for females and >33 IU for males) not fulfilling criteria for viral, alcohol-associated, or MetS-related liver disease. Participants without any of these conditions (*n* = 1499) were considered free of CLD. Finally, all CLD groups were further stratified by fibrosis stage, with advanced fibrosis defined by an AST-to-platelet ratio index (APRI) ≥ 0.7, because APRI does not include age as a component [29]. Fibrosis-4 index (FIB-4) ≥ 2.67 was additionally evaluated in sensitivity analyses [30].

### 2.4. Covariates

Several participant characteristics were included as covariates, covering demographics (age in years, continuous; sex; race/ethnicity), body mass index (BMI), lifestyle factors (smoking history, current excess alcohol use, MetS and diabetes status). Smoking history was defined as having smoked at least 100 or more cigarettes in an individual’s lifetime. Individuals with heavy or moderate current use of alcohol were labeled with a current excess alcohol use. Race/ethnicity was categorized as non-Hispanic White, non-Hispanic Black, Mexican American, or other. Cardiovascular disease and malignancy were defined by self-reported physician diagnoses. Diabetes was defined as self-reported physician-diagnosed diabetes excluding gestational diabetes or laboratory-measured glycated hemoglobin (HbA1c) ≥ 6.5% or fasting plasma glucose (FPG) ≥ 126 mg/dL or current use of insulin or oral hypoglycemic agents. Dietary intake was assessed using the NHANES dietary interview. Total daily energy intake (DRXTKCAL, kcal/day) was included as a covariate in the adjusted models. No other nutrient or dietary variables were included. Healthy physical activity was categorized according to weekly activity levels, including meeting ≥ 150 min/week of moderate-intensity activity, ≥75 min/week of vigorous-intensity activity, or ≥150 min/week of combined moderate and vigorous activity.

### 2.5. Mortality Data

Mortality status was ascertained through linkage of NHANES with the National Death Index (NDI), which provides nationwide death records maintained by the National Center for Health Statistics. Follow-up extended from the date of participation through 31 December 2019. Survivors past this date were censored at this date. Complete mortality data were available for all 2522 participants, allowing evaluation of associations between DNAm aging algorithms, CLD, and all-cause mortality.

### 2.6. Statistical Analyses

All analyses were conducted in accordance with NCHS analytic guidelines, incorporating survey weights, strata, and primary sampling units to account for the complex survey design. Participants with missing data for any variable included in the corresponding analytical model were excluded using a complete-case approach. The proportion of missing data was less than 5% for each included variable. The missing-data mechanism was not formally assessed or classified as MCAR, MAR, or MNAR. Continuous variables were examined for their distributions and plausible ranges, and no observations were excluded solely because they were statistical outliers. The primary outcomes were CLD-related phenotypes, including APRI-defined advanced fibrosis among participants with CLD, and all-cause mortality. Overall CLD status and etiology-specific analyses were considered secondary or experimental results. Baseline characteristics were summarized as weighted proportions for categorical variables and weighted means with 95% confidence intervals (CIs) for continuous variables, and compared across CLD categories. Cross-sectional associations between DNAm aging algorithms and CLD were examined using multivariable logistic regression. Odds ratios (ORs) with 95% CIs were estimated per one standard deviation (SD) increase in each DNAm aging algorithms, for overall CLD, APRI-defined advanced fibrosis, and CLD subtypes (viral, alcohol-associated, MetS-related, and uncharacterized). Models were adjusted for age, sex, smoking, excess alcohol use, metabolic syndrome, and diabetes. Longitudinal associations with all-cause mortality were assessed using Cox proportional hazards models, with time since survey participation as the time scale and censoring at death or 31 December 2019. Hazard ratios (HRs) and 95% CIs were calculated per 1-SD increase in each DNAm aging algorithms, both in the overall cohort and within CLD subset. Restricted cubic spline (RCS) analyses were performed to evaluate potential non-linear associations between DNAm aging algorithms and all-cause mortality. Time-dependent receiver operating characteristic (ROC) curve analyses were conducted to assess the discriminatory performance of DNAm aging algorithms for all-cause mortality at 1-, 10-, and 20-year follow-up intervals. Stratified analyses were additionally performed by CLD etiology, sex, and race/ethnicity to evaluate subgroup-specific associations. The proportional hazards assumption was evaluated using Schoenfeld residuals and log-minus-log survival plots. Several sensitivity analyses were performed to assess the robustness of the findings. First, advanced fibrosis was alternatively defined using FIB-4 index, and associations between DNAm aging algorithms and FIB-4-defined advanced fibrosis were examined. Second, analyses of all-cause mortality were repeated after excluding participants with a history of malignancy to evaluate potential influence from cancer-related mortality. Third, additional models were constructed by further adjusting for total daily energy intake and healthy physical activity to assess the potential impact of lifestyle-related confounding.

All statistical analyses were were carried out using R version 4.4.1 and SAS version 9.4 (SAS Institute, Inc., Cary, NC, USA), with *p* values  <  0.05 were considered statistically significant in two-sided testing after adjusting by multiple testing (false discovery rate (FDR), Benjamini–Hochberg procedure).

## 3. Results

### 3.1. Baseline Characteristics

Among the 2522 eligible participants, 1023 (40.56%) were identified with CLD. The overall study population had a mean age of 62.98 years, with females accounting for 52.57%. Etiological classification of the CLD population revealed that metabolic syndrome (MetS)-related liver diseases were the most prevalent (*n* = 441, 43.11%), followed by alcohol-related liver diseases (*n* = 316, 30.89%), liver diseases of uncharacterized etiology (*n* = 231, 22.58%), and viral liver diseases (*n* = 35, 3.42%) (Table 1). Participants with CLD exhibited distinct metabolic profiles compared to those without CLD, being older and having a higher prevalence of MetS, obesity, diabetes, and excessive alcohol use (Table A1). Additionally, unweighted analysis stratified by demographics indicated that HorvathAgeAcc and HannumAgeAcc trended upward with advancing CLD severity, whereas HorvathTelo exhibited a decreasing trend (Table A2).

### 3.2. Associations of DNAm Aging Algorithms with CLD

**Figure 2:**
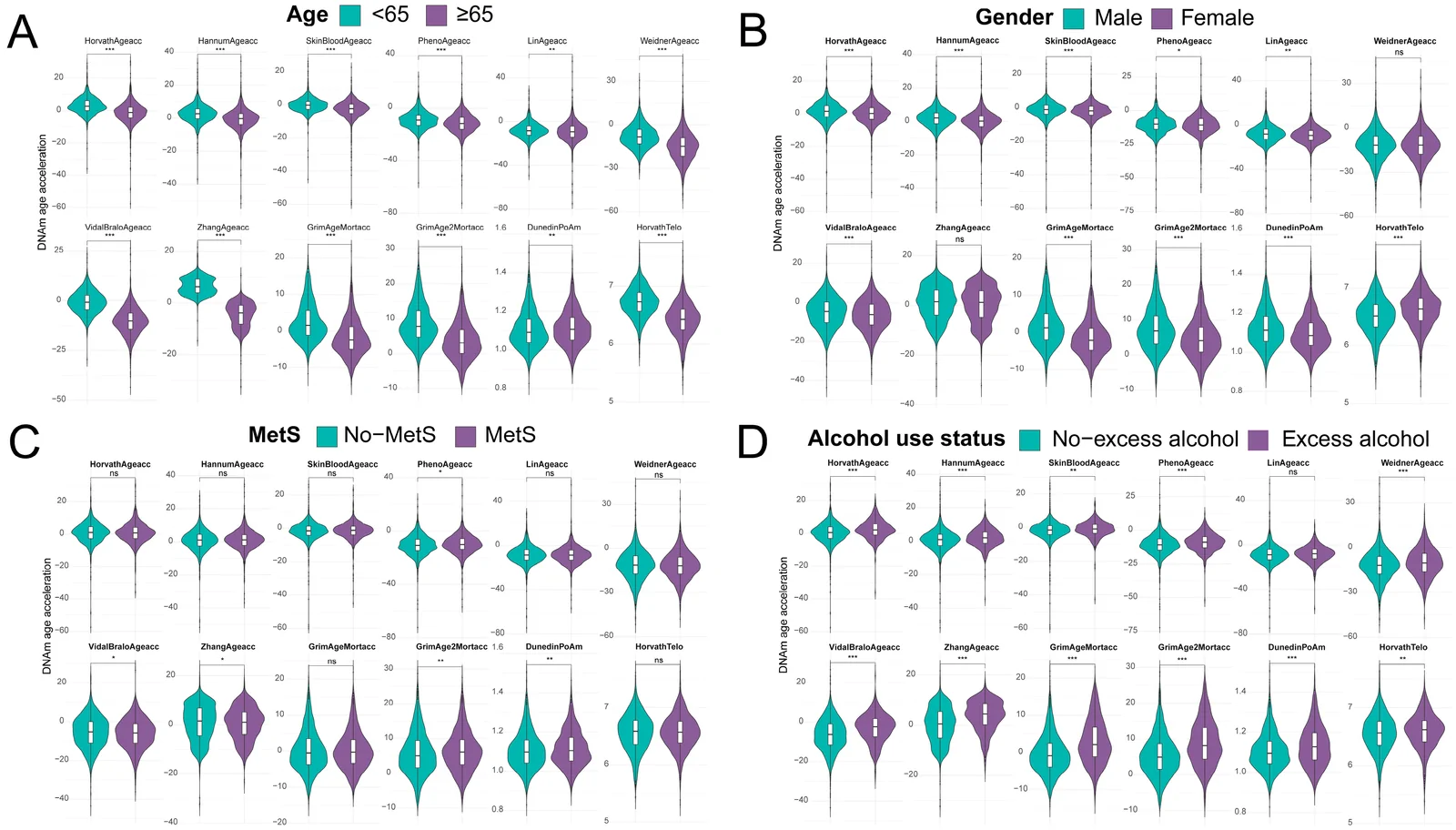
DNAm Aging Algorithms Stratified by Clinical Variables. Violin plots show the distribution of DNAm age acceleration in subgroups defined by (**A**) age, (**B**) sex, (**C**) metabolic syndrome (MetS) status, and (**D**) excessive alcohol intake. Internal box plots denote the median and interquartile range. Significance levels: ns, *p* ≥ 0.05; * *p* < 0.05; ** *p* < 0.01; *** *p* < 0.001. DNAm, DNA methylation; MetS, metabolic syndrome.

In the overall cohort, DNAm aging algorithms were not associated with CLD after multivariable adjustment (Table 2). Among participants with CLD, DunedinPoAm was nominally associated with an increased risk of APRI-defined advanced fibrosis (OR per SD = 1.28, 95% CI: 1.04–1.57), whereas HorvathTelo was nominally associated with a lower risk of APRI-defined advanced fibrosis (OR per SD = 0.65, 95% CI: 0.45–0.93). However, neither association remained significant after FDR correction (Table 2). Stratified analyses also identified several nominal associations, including inverse associations of HorvathTelo with CLD in older adults (>65 years) and females, as well as associations of Weidner-AgeAcc, GrimAgeMortAcc, and GrimAge2MortAcc among individuals without excess alcohol consumption. Nevertheless, none of these associations remained significant after FDR correction (Figure 2, Table A3).

### 3.3. Associations of DNAm Aging Algorithms with Mortality

During a median follow-up of 205 months (IQR, 111–225), 1357 deaths (53.8%) occurred. The proportional hazards assumption was satisfied for all Cox regression models based on Schoenfeld residuals and visual inspection of log-minus-log survival plots.

After adjustment for covariates, GrimAgeMortAcc, GrimAge2MortAcc, HannumAgeAcc, PhenoAgeAcc, DunedinPoAm, and HorvathTelo remained significantly associated with all-cause mortality after FDR correction (Table 3 and Table A4). GrimAge-based estimators showed the strongest positive associations across all study populations, whereas HorvathTelo was consistently associated with lower mortality risk. The strongest association was observed for PhenoAgeAcc in alcohol-associated liver disease (HR per SD = 1.63, 95% CI: 1.29–2.06).

**Figure 3:**
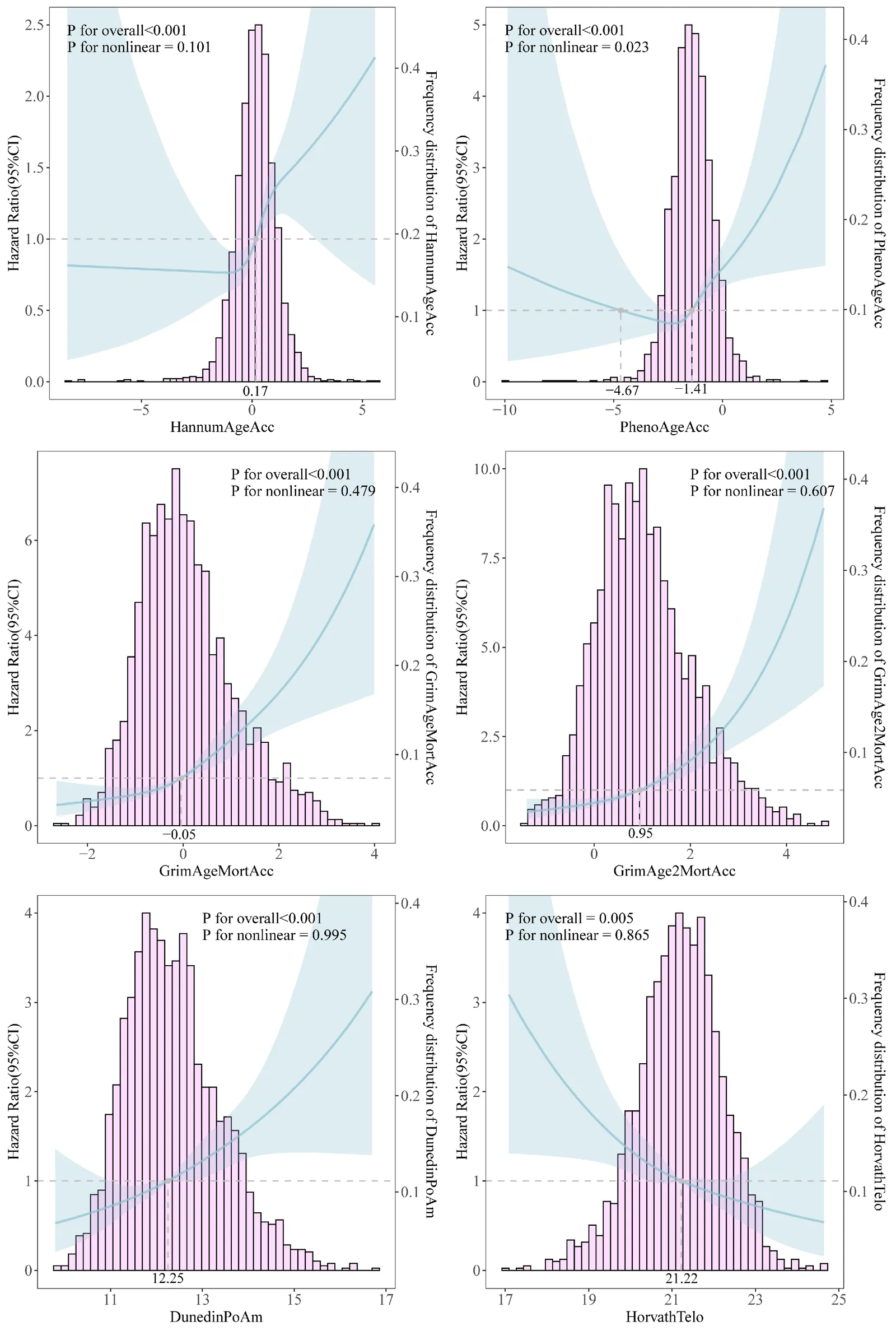
Dose–Response Associations Between DNAm Aging Algorithms and All-Cause Mortality Among Participants with CLD. Shaded areas represent 95% CIs. The horizontal dashed line indicates the reference level of HR = 1.0, and the vertical dashed line indicates the corresponding biomarker value where the RCS-estimated HR equals 1.0. *p* for nonlinear > 0.05 suggests no significant nonlinearity and indicates a linear trend. The model was adjusted for age, sex, race/ethnicity, MetS, smoking history, and presence of APRI-defined advanced fibrosis.

**Figure 4:**
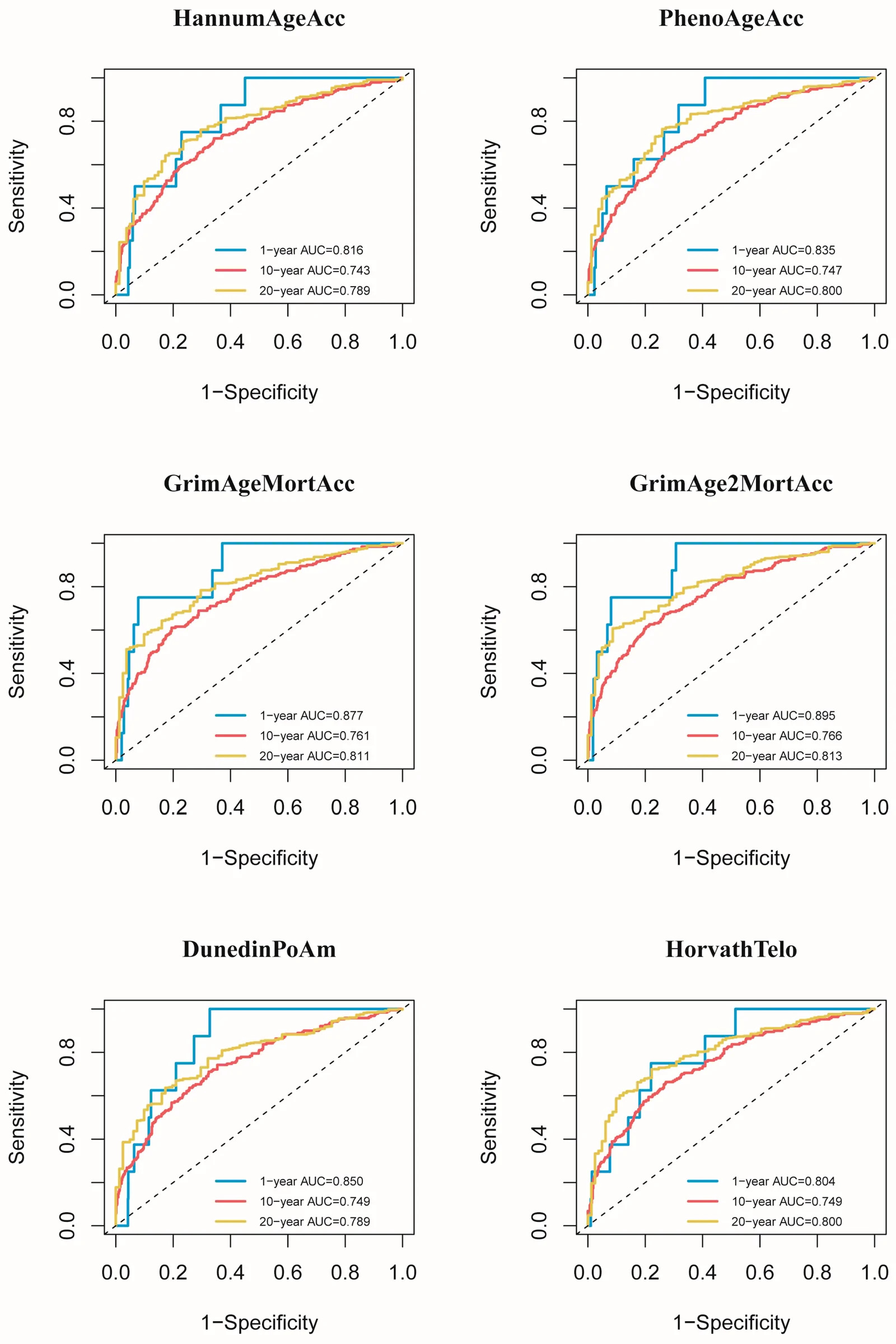
ROC Curves of DNAm Aging Algorithms in Predicting Mortality Probability Among Patients with CLD. The curves represent the discrimination performance at 1-, 10-, and 20-year follow-up, with corresponding areas under the curve (AUCs). The diagonal dashed line represents the reference line indicating no discriminative ability (AUC = 0.5). The model was adjusted for age, sex, race/ethnicity, MetS, smoking history, and presence of APRI-defined advanced fibrosis. Abbreviation: AUC, area under the curve.

RCS analysis indicated that, with the exception of PhenoAgeAcc (*p* for nonlinear = 0.023), most indicators showed a linear correlation with the risk of CLD with identified causes (Figure 3). Specifically, HorvathTelo demonstrated a negative linear correlation (*p* for overall = 0.005, *p* for nonlinear = 0.865). ROC analysis confirmed the predictive utility of these indicators. GrimAge2MortAcc demonstrated the highest predictive performance for short-, medium-, and long-term mortality (AUCs: 1 year 0.895, 10 years 0.761, 20 years 0.811), followed closely by GrimAgeMortAcc (Figure 4).

Subgroup analyses stratified by race/ethnicity further characterized these associations (Table 4 and Table A5). PhenoAgeAcc was significantly associated with mortality among non-Hispanic Whites (HR per SD = 1.39, 95% CI: 1.19–1.62) and Blacks (HR per SD = 1.27, 95% CI: 1.08–1.50), but not Mexican Americans after FDR correction. DunedinPoAm showed the strongest association among Mexican Americans (HR per SD = 1.57, 95% CI: 1.35–1.81), whereas HorvathTelo was inversely associated with mortality among Whites (HR per SD = 0.62, 95% CI: 0.50–0.76) and Blacks (HR per SD = 0.68, 95% CI: 0.52–0.88), but not other racial/ethnic groups. Similar race/ethnicity-specific associations were observed in the full cohort (Table A6).

Sex-stratified analyses showed consistent associations of GrimAgeMortAcc and GrimAge2MortAcc with mortality in both males and females (Table 5). PhenoAgeAcc and DunedinPoAm were also associated with increased mortality risk in both sexes, whereas HorvathTelo showed a significant inverse association only among females.

### 3.4. Sensitivity Analyses

As a sensitivity analysis, advanced fibrosis was alternatively defined using the FIB-4 index. The associations between DNAm aging algorithms and FIB-4-defined advanced fibrosis were partially consistent with the primary APRI-based analyses. Notably, three DNAm aging algorithms remained significantly associated with FIB-4-defined advanced fibrosis after FDR correction (Table A7).

After excluding participants with a history of malignancy (*n* = 343), the associations between DNAm aging algorithms and all-cause mortality remained largely unchanged compared with the primary analyses (Table A8, Table A9 and Table A10).

Additional adjustment for dietary factors and physical activity did not materially alter the associations between DNAm aging algorithms and all-cause mortality (Table A11 and Table A12).

## 4. Discussion

In this nationally representative cohort of U.S. adults aged ≥50 years, we evaluated the associations of DNAm aging algorithms with CLD-related phenotypes and long-term all-cause mortality. GrimAge-based DNAm aging algorithms demonstrated the most consistent and robust associations with all-cause mortality, whereas HorvathTelo showed an inverse association with all-cause mortality risk. These findings suggest that DNAm aging algorithms, particularly mortality-related estimators, may capture biological characteristics associated with long-term survival among individuals with CLD-related phenotypes.

Mounting evidence indicates that hepatic aging constitutes a pivotal driver in the progression of CLD [3], with cellular senescence triggering inflammasome activation and extracellular matrix remodeling [31]. DNAm aging algorithms have emerged as biomarkers associated with liver-related pathology beyond chronological age [32]. While prior studies have primarily relied on limited biomarkers (e.g., leukocyte telomeres) [33], our analysis systematically evaluates 12 DNAm aging algorithms in the cohort, offering evidence for their association with CLD across age, sex, MetS and excess alcohol use subgroups and all-cause mortality across sex and race/ethnicity.

Previous studies in nonalcoholic steatohepatitis (NASH) and MASLD populations have reported associations between DNAm aging algorithms and liver injury or fibrosis-related outcomes [17,34,35,36]. In our study, several DNAm aging algorithms showed nominal associations with APRI-defined advanced fibrosis among participants with CLD, whereas sensitivity analyses using FIB-4 identified significant associations for several GrimAge-based measures and HorvathTelo. These findings suggest that the relationship between DNAm aging algorithms and fibrosis-related phenotypes may vary according to the fibrosis definition applied. Overall, DNAm aging algorithms may provide biological information related to fibrosis-associated characteristics, and their relationship with fibrosis progression warrants further evaluation in longitudinal studies.

Subgroup analyses suggested potential heterogeneity in the associations between DNAm aging algorithms and CLD-related phenotypes. Nominal associations between HorvathTelo and CLD-related outcomes were mainly observed among older adults (>65 years) and females, potentially reflecting differences in cumulative aging processes and sex-specific biological factors. Previous studies have suggested that sex hormones may influence telomere maintenance and fibrosis-related responses, which may partly explain these observations [37]. In addition, nominal associations involving WeidnerAgeAcc and GrimAge-based measures varied according to alcohol consumption status, consistent with previous observations that epigenetic aging measures may capture biological aging signals differently depending on metabolic and environmental exposures [38,39]. In individuals with metabolic stress or alcohol exposure, disease-related alterations in inflammatory pathways and methylation patterns may influence the performance of DNAm aging algorithms [40,41,42]. However, the biological mechanisms underlying these subgroup-specific associations remain to be clarified.

DNAm aging algorithms are widely considered proxies for biological age associated with increased disease risk and all-cause mortality [19,43,44]. Our findings extend previous evidence by demonstrating consistent associations between DNAm aging algorithms and all-cause mortality among individuals with CLD-related phenotypes. Consistent with prior work, GrimAge-based estimators emerged as the strongest and most consistent predictors of mortality. In the original development and validation study, GrimAge predicted lifespan and healthspan with greater accuracy than earlier DNAm aging algorithms [14]. A meta-analysis of multiple cohorts also confirmed that accelerated DNAm aging algorithms, particularly HannumAgeAcc, were associated with increased mortality risk [45]. In line with these findings, we observed robust associations of GrimAgeMortAcc, GrimAge2MortAcc, HannumAgeAcc, DunedinPoAm, and HorvathTelo with all-cause mortality, independent of traditional risk factors.

Our results also align with evidence from disease-specific populations. In a study of 562 participants with underlying cardiovascular disease, HorvathAgeAcc, PhenoAgeAcc, and GrimAgeMortAcc were each significantly associated with all-cause mortality [44]. Similarly, Needham et al. demonstrated that shorter leukocyte telomere length was predictive of mortality in the U.S. adult population [46], paralleling our finding that methylation-estimated telomere length (HorvathTelo) was inversely associated with mortality risk. Interestingly, while direct telomere assays often show null or weaker associations [46,47], our results suggest that DNAm-based telomere estimates may integrate broader biological signals relevant to survival.

Our findings highlight the potential relevance of DNAm aging algorithms as molecular markers for characterizing all-cause mortality risk among individuals with CLD-related phenotypes. DNAm aging algorithms, particularly GrimAge-based estimators, may provide additional biological information beyond traditional clinical risk factors and complement existing approaches for risk assessment. Future longitudinal studies are needed to determine whether incorporating DNAm aging algorithms can improve mortality risk assessment and support personalized management of individuals with CLD.

Several limitations should be acknowledged. First, the cross-sectional design limits causal inference, and reverse causation cannot be excluded because CLD-related inflammation and metabolic alterations may influence DNAm aging estimates. Second, CLD classification was based on single biochemical measurements, and advanced fibrosis was primarily assessed using APRI with FIB-4 as a sensitivity analysis. The partially consistent findings across fibrosis definitions suggest that associations between DNAm aging algorithms and fibrosis-related phenotypes may depend on the noninvasive fibrosis assessment method. Third, the limited number of participants with viral liver disease restricted the power of etiology-specific analyses, and unavailable information on subsequent HCV treatment may affect interpretation of viral hepatitis-related findings. Fourth, the study population was restricted to adults aged ≥50 years, which may limit the generalizability of our findings. Although additional adjustment for dietary factors and physical activity did not materially alter the results, residual confounding from lifestyle factors cannot be completely excluded. Finally, although the proportion of missing data was low for all included variables, the missing-data mechanism was not formally evaluated. Therefore, the complete-case approach may have introduced some selection bias if missingness was associated with participant characteristics or study outcomes.

## 5. Conclusions

This study found that DNAm aging algorithms are strongly linked to all-cause mortality among individuals with CLD-related phenotypes. Our results indicate that biological aging, as captured by DNAm aging algorithms, is associated with all-cause mortality risk among individuals with CLD-related phenotypes, extending beyond traditional clinical factors. The observed sex- and race/ethnicity-specific pat-terns further underscore the importance of demographic context in interpreting these biomarkers. Despite the limitations of a cross-sectional design, these findings support further investigation of DNAm aging algorithms as biomarkers for characterizing all-cause mortality risk among individuals with CLD-related phenotypes. Future longitudinal studies are needed to determine whether DNAm aging algorithms can improve risk assessment and further elucidate their biological relevance in CLD.

## Figures and Tables

**Table 1:** Cohort Characteristics.

Characteristic	Complete Cohort *n* = 2522	Viral Hepatitis Liver Disease *n* = 35	Alcohol-Associated Liver Disease *n* = 316	MetS-Related Liver Disease *n* = 441	Uncharacterized Liver Disease *n* = 231
	Prop./Mean(95%CI)	Prop./Mean(95%CI)	Prop./Mean(95%CI)	Prop./Mean(95%CI)	Prop./Mean(95%CI)
Age	62.98 (62.4, 63.5)	59.36 (54.79, 63.93)	59.23 (57.92, 60.55)	64.62 (63.69, 65.56)	62.40 (60.67, 64.13)
Female	52.57 (50.1, 54.9)	51.01 (22.72, 79.29)	58.50 (50.76, 66.25)	83.19 (79.02, 87.36)	66.33 (55.62, 77.03)
Race/ethnicity					
Non-Hispanic White	66.75 (60.9, 72.5)	45.74 (17.60, 73.88)	70.05 (62.43, 77.68)	59.50 (51.93, 67.07)	68.37 (58.24, 78.50)
Non-Hispanic Black	12.45 (8.83, 16.0)	36.44 (13.50, 59.39)	8.59 (5.30, 11.89)	13.47 (8.34, 18.60)	9.20 (4.66, 13.75)
Mexican American	5.92 (3.76, 8.09)	9.89 (0.00, 21.25)	8.16 (4.34, 11.99)	7.02 (4.13, 9.91)	6.43 (4.05, 8.82)
Others	14.87 (8.88, 20.8)	7.93 (0.55, 15.31)	13.19 (7.01, 19.37)	20.01 (11.50, 28.51)	16.00 (7.98, 24.02)
BMI	28.66 (28.2, 29.0)	26.44 (23.71, 29.17)	29.21 (27.65, 30.78)	32.22 (31.30, 33.14)	25.91 (24.85, 26.98)
MetS	39.46 (36.9, 41.9)	35.68 (15.97, 55.39)	35.85 (29.55, 42.15)	100.00 (100.00, 100.00)	0.00 (0.00, 0.00)
CVD	18.32 (15.9, 20.6)	28.03 (4.13, 51.93)	15.06 (9.38, 20.73)	19.54 (14.06, 25.03)	14.56 (8.92, 20.21)
Malignancy	14.90 (13.0, 16.7)	3.12 (0.00, 7.50)	12.26 (5.82, 18.71)	13.42 (8.39, 18.46)	12.32 (7.66, 16.98)
Obese (BMI)	32.34 (29.5, 35.1)	18.61 (0.76, 36.46)	35.06 (27.68, 42.44)	53.45 (46.97, 59.94)	17.34 (9.42, 25.26)
Obese (waist circumference)	59.37 (56.2, 62.4)	41.19 (16.85, 65.53)	62.78 (55.48, 70.08)	91.65 (88.27, 95.04)	36.93 (27.08, 46.78)
Diabetes	17.26 (15.4, 19.1)	19.63 (1.85, 37.41)	15.72 (10.70, 20.75)	32.44 (27.84, 37.04)	5.45 (1.70, 9.19)
Smoking history	55.80 (52.8, 58.7)	66.05 (40.85, 91.25)	73.76 (65.78, 81.73)	37.59 (32.44, 42.75)	36.44 (28.48, 44.40)
Excess alcohol use	20.22 (17.8, 22.5)	21.46 (2.61, 40.30)	77.28 (71.33, 83.23)	0.00 (0.00, 0.00)	0.00 (0.00, 0.00)
Total daily energy intake	1907.35 (1865.15, 1949.56)	1556.84 (1141.79, 1971.89)	2059.57 (1963.30, 2155.84)	1719.39 (1617.41, 1821.38)	1817.56 (1624.69, 2010.42)
Healthy physical activity					
≥150 min/week moderate	13.90 (11.18, 16.63)	23.06 (0.00, 49.22)	14.73 (8.86, 20.59)	6.64 (2.83, 10.46)	14.23 (7.92, 20.54)
≥75 min/week vigorous	79.61 (77.05, 82.17)	55.37 (27.19, 83.56)	81.88 (75.76, 88.00)	84.96 (79.94, 89.97)	78.74 (71.21, 86.26)
150 min/week mixed (moderate + vigorous)	6.49 (5.04, 7.93)	21.57 (0.00, 45.82)	3.39 (1.26, 5.53)	8.40 (5.12, 11.67)	7.04 (2.81, 11.27)
DNAm algorithms					
HorvathAgeAcc	1.67 (1.23, 2.11)	2.20 (−0.01, 4.41)	2.70 (1.87, 3.54)	0.73 (−0.06, 1.52)	1.95 (0.79, 3.12)
HannumAgeAcc	1.39 (0.96, 1.82)	1.77 (−0.06, 3.59)	1.79 (1.23, 2.36)	0.77 (−0.17, 1.71)	1.43 (0.13, 2.73)
SkinBloodAgeAcc	−1.27 (−1.6, −0.9)	−1.67 (−3.42, 0.07)	−1.01 (−1.60, −0.43)	−1.89 (−2.67, −1.11)	−0.86 (−2.00, 0.28)
PhenoAgeAcc	−10.09 (−10.56, −9.62)	−7.09 (−8.89, −5.29)	−9.12 (−9.94, −8.29)	−10.28 (−11.45, −9.11)	−10.69 (−12.20, −9.19)
LinAgeAcc	−8.33 (−8.84, −7.81)	−7.60 (−9.04, −6.16)	−8.50 (−9.49, −7.52)	−8.75 (−9.86, −7.64)	−7.45 (−9.45, −5.45)
WeidnerAgeAcc	−10.28 (−10.93, −9.62)	−5.46 (−9.31, −1.60)	−9.39 (−11.06, −7.71)	−10.71 (−11.67, −9.76)	−9.03 (−10.76, −7.30)
VidalBraloAgeAcc	−3.63 (−4.02, −3.24)	−0.84 (−4.76, 3.08)	−1.69 (−2.94, −0.44)	−5.27 (−6.22, −4.31)	−3.17 (−4.61, −1.73)
ZhangAgeAcc	2.78 (2.38, 3.19)	4.93 (1.94, 7.92)	5.20 (4.30, 6.10)	1.62 (0.98, 2.27)	3.32 (2.04, 4.59)
GrimAgeMortAcc	0.78 (0.41, 1.15)	2.73 (−0.39, 5.85)	2.65 (1.83, 3.47)	−0.79 (−1.51, −0.07)	−1.09 (−1.99, −0.19)
GrimAge2MortAcc	6.56 (6.11, 7.01)	8.95 (5.87, 12.02)	8.62 (7.70, 9.53)	5.40 (4.56, 6.25)	4.44 (3.37, 5.50)
DunedinPoAm	1.10 (1.10, 1.11)	1.15 (1.11, 1.19)	1.12 (1.10, 1.14)	1.10 (1.09, 1.11)	1.08 (1.06, 1.10)
HorvathTelo	6.62 (6.60, 6.64)	6.67 (6.57, 6.77)	6.66 (6.62, 6.71)	6.62 (6.59, 6.65)	6.63 (6.58, 6.68)

Abbreviation: BMI, body mass index; CI, confidence interval; CVD, cardiovascular disease; DNAm, DNA methylation; MetS, metabolic dysfunction.

**Table 2:** Associations of DNAm Algorithms with Liver Disease Risk and APRI-Defined Advanced Fibrosis Risk.

DNAm Algorithms	Model OR (95% CI)	*p*-Value	FDR q-Value
CLD (1023/2522)			
HorvathAgeAcc (per SD)	1.01 (0.88, 1.16)	0.912	0.994
HannumAgeAcc (per SD)	1.00 (0.88, 1.13)	0.949	0.994
SkinBloodAgeAcc (per SD)	0.96 (0.84, 1.08)	0.462	0.917
PhenoAgeAcc (per SD)	1.00 (0.88, 1.14)	0.993	0.994
LinAgeAcc (per SD)	1.04 (0.90, 1.20)	0.603	0.917
WeidnerAgeAcc (per SD)	1.05 (0.92, 1.19)	0.476	0.917
VidalBraloAgeAcc (per SD)	0.96 (0.79, 1.16)	0.650	0.917
ZhangAgeAcc (per SD)	1.00 (0.63, 1.60)	0.994	0.994
GrimAgeMortAcc (per SD)	0.91 (0.76, 1.08)	0.271	0.812
GrimAge2MortAcc (per SD)	0.90 (0.75, 1.08)	0.247	0.812
DunedinPoAm (per SD)	1.03 (0.92, 1.16)	0.612	0.917
HorvathTelo (per SD)	0.89 (0.77, 1.04)	0.137	0.812
APRI-defined advanced fibrosis (128/1023)			
HorvathAgeAcc (per SD)	0.98 (0.77, 1.24)	0.812	0.971
HannumAgeAcc (per SD)	1.10 (0.84, 1.43)	0.482	0.971
SkinBloodAgeAcc (per SD)	0.93 (0.74, 1.17)	0.527	0.971
PhenoAgeAcc (per SD)	1.01 (0.75, 1.35)	0.971	0.971
LinAgeAcc (per SD)	0.97 (0.73, 1.29)	0.844	0.971
WeidnerAgeAcc (per SD)	1.00 (0.79, 1.26)	0.968	0.971
VidalBraloAgeAcc (per SD)	0.91 (0.63, 1.32)	0.622	0.971
ZhangAgeAcc (per SD)	1.08 (0.46, 2.50)	0.858	0.971
GrimAgeMortAcc (per SD)	1.28 (0.94, 1.76)	0.116	0.348
GrimAge2MortAcc (per SD)	1.35 (1.00, 1.82)	0.047	0.189
DunedinPoAm (per SD)	1.28 (1.04, 1.57)	0.021	0.128
HorvathTelo (per SD)	0.65 (0.45, 0.93)	0.021	0.128

Models were adjusted for age, sex, race/ethnicity, smoking history, MetS, and excess alcohol use. Abbreviation: CI, confidence interval; CLD, chronic liver disease; DNAm, DNA methylation; OR, odds ratio; SD, standard deviation.

**Table 3:** Cox Regression Survival Model in the Full Cohort and Liver Disease Etiology Subsets.

Study Population (Deaths/*n*)	All Analysis Cohort (1357/2522)	Characterized CLD (403/792)	MetS-Related Liver Disease (237/441)	Alcohol-Associated Liver Disease (145/316)
	HR (95% CI) ^1^	HR (95% CI) ^1^	HR (95% CI) ^1^	HR (95% CI) ^1^
HorvathAgeAcc (per SD)	1.24 (1.08, 1.43) ^a^	1.25 (1.09, 1.43)	1.18 (1.00, 1.40)	1.36 (1.08, 1.72) ^a^
HannumAgeAcc (per SD)	1.27 (1.16, 1.40) ^a^	1.28 (1.16, 1.42) ^a^	1.28 (1.11, 1.47) ^a^	1.28 (1.05, 1.57) ^a^
SkinBloodAgeAcc (per SD)	1.07 (0.94, 1.20)	1.06 (0.94, 1.19)	1.07 (0.93, 1.24)	0.99 (0.82, 1.19)
PhenoAgeAcc (per SD)	1.34 (1.20, 1.50) ^a^	1.33 (1.20, 1.48) ^a^	1.21 (1.05, 1.40) ^a^	1.63 (1.29, 2.06) ^a^
LinAgeAcc (per SD)	1.11 (0.99, 1.26)	1.12 (0.99, 1.27)	1.07 (0.91, 1.25)	1.16 (0.99, 1.36)
WeidnerAgeAcc (per SD)	1.04 (0.93, 1.17)	1.02 (0.89, 1.17)	1.03 (0.86, 1.25)	1.02 (0.81, 1.30)
VidalBraloAgeAcc (per SD)	1.22 (1.06, 1.41) ^a^	1.21 (1.01, 1.45)	1.23 (0.99, 1.53)	1.18 (0.85, 1.65)
ZhangAgeAcc (per SD)	1.45 (0.94, 2.24)	1.39 (0.87, 2.20)	1.48 (0.92, 2.40)	1.23 (0.38, 3.96)
GrimAgeMortAcc (per SD)	1.86 (1.62, 2.15) ^a^	1.78 (1.50, 2.11) ^a^	1.77 (1.36, 2.32) ^a^	1.87 (1.39, 2.51) ^a^
GrimAge2MortAcc (per SD)	1.89 (1.62, 2.20) ^a^	1.82 (1.52, 2.17) ^a^	1.91 (1.49, 2.45) ^a^	1.85 (1.36, 2.53) ^a^
DunedinPoAm (per SD)	1.36 (1.22, 1.52) ^a^	1.33 (1.16, 1.52) ^a^	1.27 (1.08, 1.49) ^a^	1.44 (1.21, 1.70) ^a^
HorvathTelo (per SD)	0.67 (0.60, 0.74) ^a^	0.71 (0.62, 0.82) ^a^	0.75 (0.65, 0.88) ^a^	0.63 (0.45, 0.87) ^a^

^1^ Models were adjusted for age, sex, race/ethnicity, smoking history, MetS, and presence of APRI-defined advanced fibrosis. ^a^ Remained statistically significant after false discovery rate (FDR) correction using the Benjamini–Hochberg method (FDR q-value < 0.05). Abbreviation: CI, confidence interval; CLD, chronic liver disease; HR, hazard ratio; MetS, metabolic dysfunction; SD, standard deviation.

**Table 4:** Cox Proportional Hazards Models in the Liver Disease Cohort and Race/Ethnicity Subsets.

Study Population (Deaths/*n*)	Characterized CLD (403/792)	Non-Hispanic White (155/284)	Non-Hispanic Black (98/164)	Mexican American (114/260)	Others (36/84)
	HR (95% CI)	HR (95% CI) ^1^	HR (95% CI) ^1^	HR (95% CI) ^1^	HR (95% CI) ^1^
HorvathAgeAcc (per SD)	1.25 (1.09, 1.43)	1.25 (1.06, 1.48) ^a^	1.07 (0.86, 1.33)	1.14 (0.80, 1.61)	1.23 (0.93, 1.64)
HannumAgeAcc (per SD)	1.28 (1.16, 1.42) ^a^	1.34 (1.16, 1.54) ^a^	1.30 (1.08, 1.57) ^a^	0.99 (0.75, 1.30)	1.10 (0.73, 1.66)
SkinBloodAgeAcc (per SD)	1.06 (0.94, 1.19)	1.07 (0.94, 1.23)	1.03 (0.90, 1.18)	0.78 (0.67, 0.91) ^a^	1.12 (0.86, 1.48)
PhenoAgeAcc (per SD)	1.33 (1.20, 1.48) ^a^	1.39 (1.19, 1.62) ^a^	1.27 (1.08, 1.50) ^a^	1.20 (1.01, 1.42)	1.11 (0.77, 1.61)
LinAgeAcc (per SD)	1.12 (0.99, 1.27)	1.14 (0.96, 1.34)	1.03 (0.86, 1.24)	1.12 (0.97, 1.30)	1.17 (0.77, 1.79)
WeidnerAgeAcc (per SD)	1.02 (0.89, 1.17)	1.02 (0.84, 1.24)	1.05 (0.86, 1.29)	1.10 (0.86, 1.41)	1.19 (0.84, 1.69)
VidalBraloAgeAcc (per SD)	1.21 (1.01, 1.45)	1.23 (0.99, 1.54)	1.23 (0.99, 1.51)	1.06 (0.68, 1.65)	1.07 (0.53, 2.13)
ZhangAgeAcc (per SD)	1.39 (0.87, 2.20)	1.56 (0.93, 2.59)	1.06 (0.73, 1.54)	0.57 (0.38, 0.86) ^a^	1.56 (0.52, 4.68)
GrimAgeMortAcc (per SD)	1.78 (1.50, 2.11) ^a^	2.14 (1.73, 2.65) ^a^	1.83 (1.42, 2.37) ^a^	1.27 (0.87, 1.85)	1.26 (0.73, 2.18)
GrimAge2MortAcc (per SD)	1.82 (1.52, 2.17) ^a^	2.25 (1.76, 2.88) ^a^	1.90 (1.50, 2.42) ^a^	1.45 (1.02, 2.06)	1.15 (0.71, 1.86)
DunedinPoAm (per SD)	1.33 (1.16, 1.52) ^a^	1.42 (1.19, 1.71) ^a^	1.17 (1.00, 1.38)	1.57 (1.35, 1.81) ^a^	1.14 (0.89, 1.46)
HorvathTelo (per SD)	0.71 (0.62, 0.82) ^a^	0.62 (0.50, 0.76) ^a^	0.68 (0.52, 0.88) ^a^	0.96 (0.72, 1.28)	0.89 (0.46, 1.73)

^1^ Models were adjusted for age, smoking history, MetS, and presence of APRI-defined advanced fibrosis. ^a^ Remained statistically significant after false discovery rate (FDR) correction using the Benjamini–Hochberg method (FDR q-value < 0.05). Abbreviation: CI, confidence interval; CLD, chronic liver disease; HR, hazard ratio; SD, standard deviation.

**Table 5:** Cox Proportional Hazards Models in the Liver Disease Cohort and Sex Subsets.

Study Population (Deaths/*n*)	Characterized CLD (403/792)	Male (140/258)		Female (263/534)	
	HR (95% CI)	HR (95% CI) ^1^	FDR q-Value	HR (95% CI) ^1^	FDR q-Value
HorvathAgeAcc (per SD)	1.25 (1.09, 1.43)	1.27 (0.93, 1.73)	0.256	1.22 (1.05, 1.42)	0.019
HannumAgeAcc (per SD)	1.28 (1.16, 1.42)	1.10 (0.87, 1.39)	0.508	1.35 (1.16, 1.58)	0.001
SkinBloodAgeAcc (per SD)	1.06 (0.94, 1.19)	0.97 (0.78, 1.20)	0.832	1.10 (0.92, 1.31)	0.321
PhenoAgeAcc (per SD)	1.33 (1.20, 1.48)	1.38 (1.07, 1.77)	0.042	1.31 (1.14, 1.50)	0.001
LinAgeAcc (per SD)	1.12 (0.99, 1.27)	1.09 (0.90, 1.33)	0.480	1.12 (0.97, 1.29)	0.188
WeidnerAgeAcc (per SD)	1.02 (0.89, 1.17)	0.90 (0.72, 1.13)	0.480	1.05 (0.90, 1.22)	0.539
VidalBraloAgeAcc (per SD)	1.21 (1.01, 1.45)	1.16 (0.86, 1.55)	0.480	1.15 (0.93, 1.41)	0.232
ZhangAgeAcc (per SD)	1.39 (0.87, 2.20)	1.05 (0.46, 2.36)	0.911	1.48 (0.84, 2.62)	0.223
GrimAgeMortAcc (per SD)	1.78 (1.50, 2.11)	1.76 (1.34, 2.32)	0.002	1.80 (1.48, 2.19)	<0.001
GrimAge2MortAcc (per SD)	1.82 (1.52, 2.17)	1.73 (1.30, 2.31)	0.002	1.88 (1.57, 2.26)	<0.001
DunedinPoAm (per SD)	1.33 (1.16, 1.52)	1.43 (1.19, 1.72)	0.002	1.28 (1.09, 1.50)	0.008
HorvathTelo (per SD)	0.71 (0.62, 0.82)	0.75 (0.53, 1.07)	0.256	0.74 (0.62, 0.87)	0.002

^1^ Models were adjusted for age, race/ethnicity, smoking history, MetS, and presence of APRI-defined advanced fibrosis. Abbreviation: CI, confidence interval; CLD, chronic liver disease; HR, hazard ratio; SD, standard deviation.

## Data Availability

The raw Illumina Human Methylation EPIC BeadChip array data generated in this study are available in NHANES under https://wwwn.cdc.gov/nchs/nhanes/dnam/ (accessed on 15 June 2026). The publicly available datasets analyzed in the study are available at https://wwwn.cdc.gov/nchs/nhanes/default.aspx (accessed on 15 June 2026), and are described in Section 2. Other data that support the findings of this study are available from the corresponding authors upon request.

## Appendix A

**Table A1:** Cohort Characteristics Breakdown by CLD Status.

Characteristic	Complete Cohort (*n* = 2522)	Normal Aminotransferase Levels (*n* = 1499)	CLD, No APRI-Defined Advanced Fibrosis (*n* = 703)	CLD, APRI-Defined Advanced Fibrosis (*n* = 89)
	Prop./Mean (95%CI)	Prop./Mean (95%CI)	Prop./Mean (95%CI)	Prop./Mean (95%CI)
Age	62.98 (62.4, 63.5)	62.00 (62.82, 64.31)	62.00 (61.18, 62.82)	62.73 (59.58, 65.88)
Female	52.57 (50.1, 54.9)	40.32 (36.63, 44.01)	73.02 (68.26, 77.78)	50.39 (37.92, 62.86)
Race/ethnicity				
Non-Hispanic White	66.75 (60.9, 72.5)	68.13 (61.79, 74.46)	65.00 (58.48, 71.53)	49.01 (35.47, 62.55)
Non-Hispanic Black	12.45 (8.83, 16.0)	13.17 (8.98, 17.36)	11.68 (7.53, 15.83)	16.90 (8.18, 25.62)
Mexican American	5.92 (3.76, 8.09)	4.93 (3.09, 6.77)	7.08 (4.00, 10.15)	13.76 (6.27, 21.25)
Others	14.87 (8.88, 20.8)	13.77 (7.35, 20.20)	16.24 (10.17, 22.32)	20.33 (6.46, 34.20)
BMI	28.66 (28.2, 29.0)	28.01 (27.51, 28.51)	30.73 (29.65, 31.82)	30.05 (27.51, 32.59)
MetS	39.46 (36.9, 41.9)	29.82 (26.18, 33.47)	70.65 (66.14, 75.16)	55.72 (39.96, 71.48)
CVD	18.32 (15.9, 20.6)	19.19 (16.31, 22.07)	17.22 (13.89, 20.54)	24.99 (10.04, 39.94)
Malignancy	14.90 (13.0, 16.7)	16.59 (13.99, 19.18)	12.07 (7.72, 16.41)	18.06 (5.80, 30.31)
Obese (BMI)	32.34 (29.5, 35.1)	28.47 (24.96, 31.97)	44.82 (39.41, 50.22)	36.31 (21.89, 50.74)
Obese (waist circumference)	59.37 (56.2, 62.4)	53.50 (49.82, 57.18)	77.68 (73.50, 81.86)	71.22 (59.29, 83.15)
Diabetes	17.26 (15.4, 19.1)	15.25 (13.00, 17.49)	22.74 (18.63, 26.84)	45.59 (29.41, 61.78)
Smoking history	55.80 (52.8, 58.7)	59.64 (55.74, 63.54)	52.95 (48.78, 57.12)	72.61 (60.36, 84.86)
Excess alcohol use	20.22 (17.8, 22.5)	15.65 (13.05, 18.25)	34.30 (30.02, 38.57)	41.64 (26.02, 57.25)
Total daily energy intake	1907.35 (1865.15, 1949.56)	1945.31 (1880.02, 2010.60)	1871.40 (1789.48, 1953.33)	1805.65 (1536.82, 2074.48)
Healthy physical activity				
≥150 min/week moderate	13.90 (11.18, 16.63)	15.53 (11.95, 19.10)	10.53 (7.33, 14.18)	11.53 (2.57, 19.83)
≥75 min/week vigorous	79.61 (77.05, 82.17)	78.53 (74.58, 81.75)	82.53 (79.24, 86.45)	79.53 (66.92, 92.03)
150 min/week mixed (moderate + vigorous)	6.49 (5.04, 7.93)	6.53 (4.45, 8.18)	6.53 (4.30, 8.51)	9.53 (0.00, 19.09)
DNAm algorithms				
HorvathAgeAcc	1.67 (1.23, 2.11)	1.62 (1.08, 2.17)	1.62 (1.06, 2.18)	2.07 (0.31, 3.84)
HannumAgeAcc	1.39 (0.96, 1.82)	1.45 (0.94, 1.96)	1.20 (0.62, 1.78)	1.92 (0.66, 3.19)
SkinBloodAgeAcc	−1.27 (−1.6, −0.9)	−1.22 (−1.65, −0.79)	−1.48 (−1.98, −0.98)	−1.64 (−2.89, −0.40)
PhenoAgeAcc	−10.09 (−10.56, −9.62)	−10.22 (−10.71, −9.74)	−9.66 (−10.53, −8.80)	−9.56 (−11.61, −7.50)
LinAgeAcc	−8.33 (−8.84, −7.81)	−8.32 (−8.94, −7.70)	−8.61 (−9.49, −7.72)	−8.56 (−11.02, −6.10)
WeidnerAgeAcc	−10.28 (−10.93, −9.62)	−10.66 (−11.46, −9.86)	−9.83 (−10.65, −9.01)	−11.24 (−13.66, −8.82)
VidalBraloAgeAcc	−3.63 (−4.02, −3.24)	−3.75 (−4.20, −3.31)	−3.44 (−4.23, −2.64)	−4.61 (−6.68, −2.53)
ZhangAgeAcc	2.78 (2.38, 3.19)	2.41 (1.88, 2.95)	3.35 (2.82, 3.89)	2.92 (0.63, 5.21)
GrimAgeMortAcc	0.78 (0.41, 1.15)	1.05 (0.59, 1.51)	0.69 (0.10, 1.27)	2.71 (1.02, 4.41)
GrimAge2MortAcc	6.56 (6.11, 7.01)	6.70 (6.17, 7.23)	6.78 (6.07, 7.50)	8.75 (6.97, 10.54)
DunedinPoAm	1.10 (1.10, 1.11)	1.11 (1.10, 1.11)	1.11 (1.10, 1.12)	1.14 (1.11, 1.17)
HorvathTelo	6.62 (6.60, 6.64)	6.60 (6.57, 6.63)	6.66 (6.63, 6.68)	6.51 (6.41, 6.60)

Abbreviation: BMI, body mass index; CI, confidence interval; CLD, chronic liver disease; CVD, cardiovascular disease; DNAm, DNA methylation; MetS, metabolic dysfunction.

**Table A2:** Unweighted Age-, Sex-, and MetS-Matched Comparison of Survey Participants.

**A**	**Normal Aminotransferase Levels** ***n* = 83**	**CLD,** ** No APRI-Defined Advanced Fibrosis** ***n* = 84**	**CLD,** **APRI-Defined Advanced Fibrosis** ***n* = 84**	***p* Value**	**FDR q-Value**
Age, yrs ^1^				0.999	
Mean ± sd	63.4 ± 9.9	63.5 ± 9.9	63.5 ± 9.9		
Median (Q1, Q3)	62.0 (55.0, 69.5)	62.5 (55.0, 69.2)	62.5 (55.0, 69.2)		
Female (%) ^2^	45 (54.2%)	45 (53.6%)	45 (53.6%)	1.000	
Metabolic Syndrome ^2^	52 (62.7%)	53 (63.1%)	53 (63.1%)	1.000	
DNAm algorithms (mean ± sd) ^1^					
HorvathAgeAcc	0.23 ± 7.04	1.47 ± 5.74	1.66 ± 5.31	0.435	0.603
HannumAgeAcc	−0.11 ± 6.35	1.22 ± 5.22	2.15 ± 5.51	0.049	0.294
SkinBloodAgeAcc	−2.35 ± 5.68	−1.74 ± 5.14	−1.37 ± 4.44	0.452	0.603
PhenoAgeAcc	−10.67 ± 6.98	−9.26 ± 7.54	−8.62 ± 6.34	0.132	0.396
LinAgeAcc	−10.11 ± 8.30	−7.45 ± 7.99	−8.76 ± 6.65	0.123	0.396
WeidnerAgeAcc	−11.24 ± 8.07	−9.22 ± 9.69	−11.16 ± 7.96	0.235	0.470
VidalBraloAgeAcc	−5.15 ± 7.15	−4.12 ± 6.98	−5.49 ± 7.03	0.419	0.603
ZhangAgeAcc	2.01 ± 6.89	2.32 ± 6.64	2.41 ± 6.78	0.921	0.921
GrimAgeMortAcc	1.31 ± 5.45	1.28 ± 5.27	2.15 ± 5.85	0.644	0.703
GrimAge2MortAcc	7.56 ± 5.81	7.42 ± 5.69	8.44 ± 6.28	0.597	0.703
DunedinPoAm	1.11 ± 0.09	1.12 ± 0.09	1.14 ± 0.09	0.205	0.470
HorvathTelo	6.67 ± 0.29	6.60 ± 0.25	6.52 ± 0.30	0.005	0.060
**B**	**No APRI-Defined Advanced Fibrosis** ***n* = 84**	**CLD, APRI-Defined Advanced Fibrosis** ***n* = 84**		***p* Value**	**FDR q-Value**
Age, yrs ^1^				1.000	
mean ± sd	63.5 ± 9.9	63.5 ± 9.9			
Median (Q1, Q3)	62.5 (55.0, 69.2)	62.5 (55.0, 69.2)			
Female (%) ^2^	45 (53.6%)	45 (53.6%)		1.000	
Metabolic Syndrome ^2^	53 (63.1%)	53 (63.1%)		1.000	
DNAm algorithms (mean ± sd) ^1^					
HorvathAgeAcc	0.33 ± 6.25	1.66 ± 5.31		0.203	0.338
HannumAgeAcc	0.51 ± 5.96	2.15 ± 5.51		0.056	0.168
SkinBloodAgeAcc	−2.12 ± 4.88	−1.37 ± 4.44		0.226	0.338
PhenoAgeAcc	−10.11 ± 7.26	−8.62 ± 6.34		0.138	0.276
LinAgeAcc	−8.73 ± 7.82	−8.76 ± 6.65		0.976	0.976
WeidnerAgeAcc	−9.59 ± 8.29	−11.16 ± 7.96		0.391	0.469
VidalBraloAgeAcc	−5.22 ± 6.90	−5.49 ± 7.03		0.855	0.933
ZhangAgeAcc	2.13 ± 6.72	2.41 ± 6.78		0.743	0.892
GrimAgeMortAcc	0.56 ± 4.85	2.15 ± 5.85		0.159	0.276
GrimAge2MortAcc	6.69 ± 5.16	8.44 ± 6.28		0.112	0.269
DunedinPoAm	1.11 ± 0.09	1.14 ± 0.09		0.053	0.168
HorvathTelo	6.65 ± 0.28	6.52 ± 0.30		0.007	0.084

Panel A—comparison of all three groups (normal aminotransferase, liver disease without advanced fibrosis, and advanced fibrosis. Panel B—comparison of advanced fibrosis groups vs. combination of normal aminotransferase and liver disease without advanced fibrosis. ^1^ Kruskal–Wallis rank sum test; ^2^ Pearson’s Chi-squared test. Abbreviation: DNAm, DNA methylation; sd, standard deviation; CLD, chronic liver disease.

**Table A3:** Associations of DNAm Algorithm Acceleration Biomarkers with Liver Disease Risk in the Study Population by Age, Sex, MetS, and Excess Alcohol Use Status.

**A**	**Aged 50–65 Years** **(*n* = 1274)**	***p*-Value**	**FDR q-Value**	**Aged > 65 Years** **(*n* = 1248)**	***p*-Value**	**FDR q-Value**
**DNAm** **Algorithms**	**OR (95% CI)**			**OR (95% CI)**		
HorvathAgeAcc (per SD)	1.00 (0.80, 1.25)	0.993	0.993	1.00 (0.85, 1.18)	0.984	0.984
HannumAgeAcc (per SD)	0.94 (0.76, 1.16)	0.529	0.993	0.94 (0.94, 1.25)	0.270	0.463
SkinBloodAgeAcc (per SD)	0.99 (0.81, 1.22)	0.914	0.993	0.99 (0.79, 1.05)	0.180	0.432
PhenoAgeAcc (per SD)	1.02 (0.86, 1.21)	0.792	0.993	1.02 (0.82, 1.18)	0.847	0.984
LinAgeAcc (per SD)	0.96 (0.79, 1.16)	0.629	0.993	0.96 (0.96, 1.40)	0.122	0.432
WeidnerAgeAcc (per SD)	1.12 (0.96, 1.31)	0.134	0.993	1.12 (0.75, 1.18)	0.566	0.849
VidalBraloAgeAcc (per SD)	1.04 (0.77, 1.41)	0.786	0.993	1.04 (0.65, 1.09)	0.183	0.432
ZhangAgeAcc (per SD)	1.00 (0.49, 2.03)	0.991	0.993	1.00 (0.56, 1.74)	0.965	0.984
GrimAgeMortAcc (per SD)	0.93 (0.75, 1.16)	0.512	0.993	0.93 (0.71, 1.08)	0.216	0.432
GrimAge2MortAcc (per SD)	0.93 (0.76, 1.15)	0.509	0.993	0.93 (0.69, 1.08)	0.193	0.432
DunedinPoAm (per SD)	1.03 (0.88, 1.20)	0.699	0.993	1.03 (0.88, 1.22)	0.675	0.899
HorvathTelo (per SD)	0.93 (0.74, 1.16)	0.507	0.993	0.93 (0.71, 0.99)	0.034	0.407
**B**	**Males** **(*n* = 1282)**	***p*-Value**	**FDR q-Value**	**Females** **(*n* = 1240)**	***p*-Value**	**FDR q-Value**
**DNAm Algorithms**	**OR (95% CI)**			**OR (95% CI)**		
HorvathAgeAcc (per SD)	1.04 (0.86, 1.27)	0.661	0.955	0.97 (0.83, 1.14)	0.704	0.845
HannumAgeAcc (per SD)	0.99 (0.79, 1.26)	0.956	0.956	0.98 (0.86, 1.12)	0.795	0.874
SkinBloodAgeAcc (per SD)	1.06 (0.89, 1.27)	0.503	0.955	0.88 (0.77, 1.01)	0.060	0.360
PhenoAgeAcc (per SD)	1.07 (0.84, 1.36)	0.567	0.955	0.97 (0.81, 1.15)	0.681	0.845
LinAgeAcc (per SD)	1.02 (0.86, 1.21)	0.847	0.956	1.04 (0.87, 1.25)	0.667	0.845
WeidnerAgeAcc (per SD)	0.95 (0.80, 1.13)	0.554	0.955	1.08 (0.88, 1.32)	0.455	0.809
VidalBraloAgeAcc (per SD)	0.97 (0.69, 1.36)	0.848	0.956	0.95 (0.76, 1.18)	0.608	0.845
ZhangAgeAcc (per SD)	1.23 (0.64, 2.36)	0.531	0.955	0.82 (0.51, 1.34)	0.423	0.809
GrimAgeMortAcc (per SD)	0.87 (0.70, 1.08)	0.192	0.955	0.93 (0.73, 1.17)	0.507	0.809
GrimAge2MortAcc (per SD)	0.89 (0.72, 1.09)	0.255	0.955	0.90 (0.70, 1.17)	0.425	0.809
DunedinPoAm (per SD)	1.04 (0.87, 1.24)	0.687	0.955	0.99 (0.84, 1.17)	0.908	0.908
HorvathTelo (per SD)	1.05 (0.84, 1.31)	0.655	0.955	**0.78 (0.64, 0.94)**	**0.013**	0.152
**C**	**No-Mets Diagnosis** **(*n* = 1444)**	***p*-Value**	**FDR q-Value**	**Mets Diagnosis** **(*n* = 1078))**	***p*-Value**	**FDR q-Value**
**DNAm Algorithms**	**OR (95% CI)**			**OR (95% CI**		
HorvathAgeAcc (per SD)	1.11 (0.95, 1.29)	0.180	0.991	0.91 (0.74, 1.12)	0.357	0.714
HannumAgeAcc (per SD)	1.00 (0.86, 1.17)	0.989	0.991	1.04 (0.85, 1.26)	0.715	0.953
SkinBloodAgeAcc (per SD)	1.00 (0.88, 1.13)	0.991	0.991	0.94 (0.76, 1.17)	0.576	0.863
PhenoAgeAcc (per SD)	1.02 (0.87, 1.19)	0.813	0.991	1.01 (0.81, 1.25)	0.962	0.962
LinAgeAcc (per SD)	1.02 (0.88, 1.18)	0.775	0.991	1.09 (0.86, 1.40)	0.464	0.795
WeidnerAgeAcc (per SD)	1.01 (0.88, 1.16)	0.893	0.991	1.12 (0.91, 1.37)	0.275	0.680
VidalBraloAgeAcc (per SD)	1.08 (0.89, 1.31)	0.440	0.991	0.84 (0.62, 1.13)	0.233	0.680
ZhangAgeAcc (per SD)	1.06 (0.65, 1.75)	0.804	0.991	1.08 (0.56, 2.08)	0.817	0.962
GrimAgeMortAcc (per SD)	0.91 (0.72, 1.14)	0.387	0.991	0.87 (0.66, 1.13)	0.284	0.680
GrimAge2MortAcc (per SD)	0.91 (0.73, 1.15)	0.422	0.991	0.84 (0.64, 1.10)	0.195	0.680
DunedinPoAm (per SD)	1.01 (0.88, 1.17)	0.841	0.991	1.01 (0.82, 1.25)	0.899	0.962
HorvathTelo (per SD)	0.93 (0.74, 1.17)	0.525	0.991	0.79 (0.62, 1.01)	0.056	0.677
**D**	**No-Excess Alcohol** **(*n* = 2054)**	***p*-Value**	**FDR q-Value**	**Excess Alcohol** **(*n* = 468)**	***p*-Value**	**FDR q-Value**
**DNAm Algorithms**	**OR (95% CI)**			**OR (95% CI)**		
HorvathAgeAcc (per SD)	1.03 (0.88, 1.21)	0.698	0.917	1.00 (0.77, 1.31)	0.981	0.981
HannumAgeAcc (per SD)	1.01 (0.86, 1.19)	0.865	0.926	1.01 (0.79, 1.29)	0.944	0.981
SkinBloodAgeAcc (per SD)	0.98 (0.82, 1.16)	0.764	0.917	0.94 (0.75, 1.18)	0.584	0.981
PhenoAgeAcc (per SD)	0.97 (0.84, 1.13)	0.698	0.917	1.15 (0.92, 1.42)	0.211	0.981
LinAgeAcc (per SD)	1.07 (0.91, 1.26)	0.426	0.917	0.97 (0.71, 1.31)	0.817	0.981
WeidnerAgeAcc (per SD)	1.13 (1.02, 1.25)	0.026	0.104	0.94 (0.70, 1.26)	0.648	0.981
VidalBraloAgeAcc (per SD)	0.97 (0.78, 1.20)	0.745	0.917	1.03 (0.75, 1.42)	0.848	0.981
ZhangAgeAcc (per SD)	1.12 (0.60, 2.09)	0.716	0.917	0.90 (0.37, 2.18)	0.801	0.981
GrimAgeMortAcc (per SD)	0.80 (0.67, 0.96)	0.020	0.104	1.08 (0.78, 1.50)	0.624	0.981
GrimAge2MortAcc (per SD)	0.80 (0.66, 0.95)	0.014	0.104	1.10 (0.78, 1.54)	0.587	0.981
DunedinPoAm (per SD)	0.99 (0.87, 1.14)	0.926	0.926	1.07 (0.84, 1.35)	0.575	0.981
HorvathTelo (per SD)	0.87 (0.74, 1.02)	0.090	0.271	0.94 (0.71, 1.23)	0.623	0.981

Panel A—Models were adjusted for sex, Mets, smoking history and excess alcohol use. Panel B—Models were adjusted for age, Mets, smoking history and excess alcohol use. Panel C—Models were adjusted for age, sex, smoking history and excess alcohol use. Panel D—Models were adjusted for age, sex, Mets and smoking history. Abbreviation: CI, confidence interval; DNAm, DNA methylation; MetS, metabolic dysfunction; OR, odds Ratio; SD, standard deviation.

**Table A4:** False Discovery Rate Correction for Table 3: Cox Proportional Hazards Models in the Full Cohort and Liver Disease Etiology Subsets.

Study Population (Deaths/*n*)	All Analysis Cohort (1357/2522)		Characterized CLD (403/792)		MetS-Related Liver Disease (237/441)		Alcohol-Associated Liver Disease (145/316)	
	*p*-Value	FDR q-Value	*p*-Value	FDR q-Value	*p*-Value	FDR q-Value	*p*-Value	FDR q-Value
HorvathAgeAcc (per SD)	0.004	0.007	0.002	0.003	0.056	0.094	0.011	0.022
HannumAgeAcc (per SD)	<0.001	<0.001	<0.001	<0.001	0.001	0.004	0.017	0.029
SkinBloodAgeAcc (per SD)	0.286	0.312	0.315	0.344	0.315	0.378	0.875	0.875
PhenoAgeAcc (per SD)	<0.001	<0.001	<0.001	<0.001	0.012	0.024	<0.001	0.001
LinAgeAcc (per SD)	0.075	0.100	0.061	0.082	0.403	0.439	0.074	0.111
WeidnerAgeAcc (per SD)	0.492	0.492	0.772	0.772	0.715	0.715	0.839	0.875
VidalBraloAgeAcc (per SD)	0.008	0.012	0.044	0.066	0.063	0.094	0.316	0.422
ZhangAgeAcc (per SD)	0.093	0.111	0.159	0.191	0.106	0.142	0.718	0.862
GrimAgeMortAcc (per SD)	<0.001	<0.001	<0.001	<0.001	<0.001	0.001	<0.001	0.001
GrimAge2MortAcc (per SD)	<0.001	<0.001	<0.001	<0.001	<0.001	<0.001	<0.001	0.001
DunedinPoAm (per SD)	<0.001	<0.001	<0.001	<0.001	0.006	0.015	<0.001	0.001
HorvathTelo (per SD)	<0.001	<0.001	<0.001	<0.001	0.001	0.003	0.006	0.015

Models were adjusted for age, sex, race/ethnicity, smoking history, MetS, and presence of advanced fibrosis. Abbreviation: CLD, chronic liver disease; MetS, metabolic dysfunction; SD, standard deviation.

**Table A5:** False Discovery Rate Correction for Table 4: Cox Proportional Hazards Models in the Liver Disease Cohort and Race/Ethnicity Subsets.

Study Population (Deaths/*n*)	Non-Hispanic White (155/284)		Non-Hispanic Black (98/164)		Mexican American (114/260)		Others (36/84)	
	*p*-Value	FDR q-Value	*p*-Value	FDR q-Value	*p*-Value	FDR q-Value	*p*-Value	FDR q-Value
HorvathAgeAcc (per SD)	0.010	0.018	0.510	0.757	0.454	0.605	0.146	0.760
HannumAgeAcc (per SD)	<0.001	0.001	0.008	0.020	0.926	0.926	0.633	0.760
SkinBloodAgeAcc (per SD)	0.282	0.307	0.635	0.757	0.003	0.015	0.390	0.760
PhenoAgeAcc (per SD)	<0.001	<0.001	0.006	0.018	0.036	0.092	0.576	0.760
LinAgeAcc (per SD)	0.137	0.165	0.737	0.757	0.117	0.235	0.456	0.760
WeidnerAgeAcc (per SD)	0.821	0.821	0.621	0.757	0.430	0.605	0.313	0.760
VidalBraloAgeAcc (per SD)	0.066	0.098	0.058	0.100	0.795	0.867	0.853	0.853
ZhangAgeAcc (per SD)	0.087	0.116	0.757	0.757	0.008	0.033	0.413	0.760
GrimAgeMortAcc (per SD)	<0.001	<0.001	<0.001	<0.001	0.199	0.342	0.391	0.760
GrimAge2MortAcc (per SD)	<0.001	<0.001	<0.001	<0.001	0.038	0.092	0.567	0.760
DunedinPoAm (per SD)	<0.001	0.001	0.053	0.100	<0.001	<0.001	0.287	0.760
HorvathTelo (per SD)	<0.001	<0.001	0.005	0.018	0.770	0.867	0.724	0.790

Models were adjusted for age, sex, smoking history, MetS. Abbreviation: SD, standard deviation.

**Table A6:** Cox Proportional Hazards Models in the Full Cohort and Race/Ethnicity Subsets.

Study Population (Deaths/*n*)	All Analysis Cohort (1357/2522)	Non-Hispanic White (602/1024)	Non-Hispanic Black (310/537)	Mexican American (345/715)	Others (100/246)
	HR (95% CI)	HR (95% CI) ^1^	HR (95% CI) ^1^	HR (95% CI) ^1^	HR (95% CI) ^1^
HorvathAgeAcc (per SD)	1.24 (1.08, 1.43)	1.16 (1.05, 1.28)	1.19 (1.04, 1.37)	1.20 (0.98, 1.46)	1.04 (0.88, 1.21)
HannumAgeAcc (per SD)	1.27 (1.16, 1.40)	1.27 (1.16, 1.40)	1.31 (1.16, 1.48)	1.15 (1.01, 1.31)	1.16 (0.86, 1.24)
SkinBloodAgeAcc (per SD)	1.07 (0.94, 1.20)	1.11 (0.99, 1.23)	1.11 (1.00, 1.23)	0.96 (0.83, 1.11)	1.00 (0.85, 1.09)
PhenoAgeAcc (per SD)	1.34 (1.20, 1.50)	1.35 (1.19, 1.54)	1.37 (1.21, 1.56)	1.25 (1.11, 1.40)	1.21 (0.90, 1.30)
LinAgeAcc (per SD)	1.11 (0.99, 1.26)	1.08 (1.00, 1.17)	1.18 (1.02, 1.35)	1.15 (1.00, 1.32)	1.02 (0.73, 1.03)
WeidnerAgeAcc (per SD)	1.04 (0.93, 1.17)	1.05 (0.96, 1.16)	1.00 (0.91, 1.10)	1.13 (0.96, 1.34)	0.91 (0.74, 1.48)
VidalBraloAgeAcc (per SD)	1.22 (1.06, 1.41)	1.20 (1.06, 1.36)	1.24 (1.02, 1.50)	1.21 (1.01, 1.46)	1.02 (0.74, 1.67)
ZhangAgeAcc (per SD)	1.45 (0.94, 2.24)	1.65 (1.08, 2.52)	1.42 (0.95, 2.13)	1.12 (0.73, 1.71)	0.95 (0.64, 2.14)
GrimAgeMortAcc (per SD)	1.86 (1.62, 2.15)	1.85 (1.62, 2.11)	1.56 (1.37, 1.79)	1.30 (1.12, 1.51)	1.37 (1.12, 1.95)
GrimAge2MortAcc (per SD)	1.89 (1.62, 2.20)	1.86 (1.61, 2.15)	1.69 (1.45, 1.96)	1.40 (1.18, 1.66)	1.45 (1.13, 1.75)
DunedinPoAm (per SD)	1.36 (1.22, 1.52)	1.38 (1.25, 1.53)	1.26 (1.12, 1.41)	1.34 (1.18, 1.51)	1.12 (0.91, 1.40)
HorvathTelo (per SD)	0.67 (0.60, 0.74)	0.69 (0.61, 0.79)	0.66 (0.54, 0.80)	0.85 (0.72, 1.00)	0.54 (0.48, 1.10)

^1^ Models were adjusted for age, sex, smoking history, MetS. Abbreviation: CI, confidence interval; HR, hazard Ratio; SD, standard deviation.

**Table A7:** Sensitivity Analysis Using APRI- and FIB-4-Defined Advanced Fibrosis.

DNAm Algorithms	APRI-Defined Advanced Fibrosis (128/1023)			FIB-4-Defined Advanced Fibrosis (86/1023)		
	Model OR (95% CI)	*p*-Value	FDR q-Value	Model OR (95% CI)	*p*-Value	FDR q-Value
HorvathAgeAcc (per SD)	0.98 (0.77, 1.24)	0.812	0.971	1.07 (0.82, 1.39)	0.615	0.719
HannumAgeAcc (per SD)	1.10 (0.84, 1.43)	0.482	0.971	1.07 (0.77, 1.49)	0.659	0.719
SkinBloodAgeAcc (per SD)	0.93 (0.74, 1.17)	0.527	0.971	1.00 (0.73, 1.37)	0.990	0.990
PhenoAgeAcc (per SD)	1.01 (0.75, 1.35)	0.971	0.971	1.22 (0.95, 1.58)	0.111	0.266
LinAgeAcc (per SD)	0.97 (0.73, 1.29)	0.844	0.971	1.22 (0.85, 1.76)	0.263	0.433
WeidnerAgeAcc (per SD)	1.00 (0.79, 1.26)	0.968	0.971	1.37 (1.00, 1.88)	0.041	0.123
VidalBraloAgeAcc (per SD)	0.91 (0.63, 1.32)	0.622	0.971	1.10 (0.77, 1.59)	0.580	0.719
ZhangAgeAcc (per SD)	1.08 (0.46, 2.50)	0.858	0.971	2.10 (0.57, 7.71)	0.242	0.433
GrimAgeMortAcc (per SD)	1.28 (0.94, 1.76)	0.116	0.348	1.74 (1.14, 2.65)	0.007	0.030
GrimAge2MortAcc (per SD)	1.35 (1.00, 1.82)	0.047	0.189	1.76 (1.17, 2.65)	0.005	0.028
DunedinPoAm (per SD)	1.28 (1.04, 1.57)	0.021	0.128	1.21 (0.84, 1.74)	0.289	0.433
HorvathTelo (per SD)	0.65 (0.45, 0.93)	0.021	0.128	0.55 (0.37, 0.83)	0.003	0.028

Models were adjusted for age, sex, race/ethnicity, smoking history, MetS, and excess alcohol use. Abbreviation: CI, confidence interval; DNAm, DNA methylation; OR, odds ratio; SD, standard deviation.

**Table A8:** Cox Proportional Hazards Models for All-Cause Mortality After Excluding Participants with Malignancy.

Study Population (Deaths/*n*)	Characterized CLD (348/706)		MetS-Related Liver Disease (202/389)		Alcohol-Associated Liver Disease (127/284)	
DNAm Algorithms	HR (95% CI)	FDR q-Value	HR (95% CI) ^1^	FDR q-Value	HR (95% CI) ^1^	FDR q-Value
HorvathAgeAcc (per SD)	1.21 (1.05, 1.39)	0.021	1.07 (0.88, 1.30)	0.576	1.47 (1.14, 1.90)	0.011
HannumAgeAcc (per SD)	1.22 (1.10, 1.35)	0.001	1.20 (1.03, 1.39)	0.064	1.26 (0.99, 1.60)	0.088
SkinBloodAgeAcc (per SD)	1.03 (0.92, 1.16)	0.608	1.03 (0.89, 1.19)	0.756	0.98 (0.78, 1.24)	0.882
PhenoAgeAcc (per SD)	1.35 (1.21, 1.51)	<0.001	1.18 (0.97, 1.42)	0.155	1.73 (1.27, 2.37)	0.005
LinAgeAcc (per SD)	1.08 (0.95, 1.23)	0.256	0.99 (0.84, 1.18)	0.937	1.20 (0.99, 1.44)	0.088
WeidnerAgeAcc (per SD)	1.09 (0.95, 1.24)	0.256	1.08 (0.88, 1.32)	0.576	1.10 (0.86, 1.42)	0.521
VidalBraloAgeAcc (per SD)	1.26 (1.01, 1.56)	0.057	1.26 (0.96, 1.64)	0.155	1.25 (0.87, 1.81)	0.293
ZhangAgeAcc (per SD)	1.27 (0.79, 2.04)	0.341	1.32 (0.81, 2.16)	0.383	1.10 (0.29, 4.17)	0.882
GrimAgeMortAcc (per SD)	1.69 (1.41, 2.02)	<0.001	1.67 (1.20, 2.31)	0.020	1.80 (1.33, 2.42)	0.005
GrimAge2MortAcc (per SD)	1.73 (1.43, 2.09)	<0.001	1.80 (1.31, 2.49)	0.010	1.77 (1.28, 2.45)	0.005
DunedinPoAm (per SD)	1.26 (1.09, 1.45)	0.005	1.20 (1.00, 1.46)	0.131	1.35 (1.11, 1.63)	0.010
HorvathTelo (per SD)	0.72 (0.63, 0.81)	<0.001	0.74 (0.59, 0.93)	0.052	0.67 (0.46, 0.98)	0.076

^1^ Models were adjusted for age, sex, race/ethnicity, smoking history, MetS, and presence of APRI-defined advanced fibrosis. Abbreviation: CI, confidence interval; CLD, chronic liver disease; DNAm, DNA methylation; HR, hazard ratio; MetS, metabolic dysfunction; SD, standard deviation.

**Table A9:** Sensitivity Analysis of Cox Regression Survival Models in the Characterized Liver Disease Cohort and Race/Ethnicity Subgroups After Excluding Participants with Malignancy.

Study Population (Deaths/*n*)	Non-Hispanic White (121/230)		Non-Hispanic Black (88/152)		Mexican American (105/243)		Others (34/81)	
DNAm Algorithms	HR (95% CI)	FDR q-Value	HR (95% CI)	FDR q-Value	HR (95% CI)	FDR q-Value	HR (95% CI)	FDR q-Value
HorvathAgeAcc (per SD)	1.25 (1.06–1.48)	0.018	1.07 (0.86–1.33)	0.757	1.14 (0.80–1.61)	0.605	1.23 (0.93–1.64)	0.760
HannumAgeAcc (per SD)	1.34 (1.15–1.54)	0.001	1.30 (1.08–1.57)	0.020	0.99 (0.75–1.30)	0.926	1.10 (0.73–1.66)	0.760
SkinBloodAgeAcc (per SD)	1.07 (0.94–1.23)	0.307	1.03 (0.90–1.18)	0.757	0.78 (0.67–0.91)	0.015	1.12 (0.85–1.48)	0.760
PhenoAgeAcc (per SD)	1.39 (1.19–1.62)	<0.001	1.27 (1.08–1.50)	0.018	1.20 (1.01–1.42)	0.092	1.11 (0.76–1.61)	0.760
LinAgeAcc (per SD)	1.13 (0.96–1.34)	0.165	1.03 (0.86–1.24)	0.757	1.12 (0.97–1.30)	0.235	1.17 (0.77–1.78)	0.760
WeidnerAgeAcc (per SD)	1.02 (0.84–1.24)	0.821	1.05 (0.86–1.29)	0.757	1.10 (0.86–1.41)	0.605	1.19 (0.84–1.69)	0.760
VidalBraloAgeAcc (per SD)	1.23 (0.99–1.54)	0.098	1.23 (0.99–1.51)	0.100	1.06 (0.68–1.64)	0.867	1.07 (0.53–2.13)	0.853
ZhangAgeAcc (per SD)	1.56 (0.93–2.59)	0.116	1.06 (0.73–1.54)	0.757	0.57 (0.38–0.86)	0.033	1.56 (0.52–4.68)	0.760
GrimAgeMortAcc (per SD)	2.14 (1.73–2.65)	<0.001	1.83 (1.42–2.37)	<0.001	1.27 (0.87–1.85)	0.342	1.26 (0.73–2.18)	0.760
GrimAge2MortAcc (per SD)	2.25 (1.76–2.88)	<0.001	1.90 (1.50–2.42)	<0.001	1.45 (1.02–2.06)	0.092	1.15 (0.71–1.86)	0.760
DunedinPoAm (per SD)	1.42 (1.19–1.71)	0.001	1.17 (1.00–1.38)	0.100	1.57 (1.35–1.81)	<0.001	1.14 (0.89–1.46)	0.760
HorvathTelo (per SD)	0.62 (0.50–0.76)	<0.001	0.68 (0.52–0.88)	0.018	0.96 (0.72–1.28)	0.867	0.89 (0.46–1.73)	0.790

Models were adjusted for age, sex, smoking history, MetS, and presence of APRI-defined advanced fibrosis. Abbreviation: CI, confidence interval; DNAm, DNA methylation; HR, hazard ratio; SD, standard deviation.

**Table A10:** Sensitivity Analysis of Cox Regression Survival Models in the Characterized Liver Disease Cohort and Sex Subgroups After Excluding Participants with Malignancy.

Study Population (Deaths/*n*)	Characterized CLD (348/706)		Male (126/239)		Female (222/467)	
DNAm Algorithms	HR (95% CI)	FDR q-Value	HR (95% CI) ^1^	FDR q-Value	HR (95% CI) ^1^	FDR q-Value
HorvathAgeAcc (per SD)	1.21 (1.05, 1.39)	0.021	1.25 (0.93–1.69)	0.276	1.16 (0.98–1.38)	0.128
HannumAgeAcc (per SD)	1.22 (1.10, 1.35)	0.001	1.03 (0.84–1.27)	0.823	1.29 (1.07–1.54)	0.023
SkinBloodAgeAcc (per SD)	1.03 (0.92, 1.16)	0.608	0.94 (0.76–1.17)	0.751	1.07 (0.89–1.29)	0.481
PhenoAgeAcc (per SD)	1.35 (1.21, 1.51)	<0.001	1.35 (1.07–1.72)	0.044	1.34 (1.14–1.58)	0.004
LinAgeAcc (per SD)	1.08 (0.95, 1.23)	0.256	1.01 (0.84–1.22)	0.893	1.10 (0.94–1.29)	0.263
WeidnerAgeAcc (per SD)	1.09 (0.95, 1.24)	0.256	0.94 (0.74–1.18)	0.751	1.13 (0.97–1.31)	0.182
VidalBraloAgeAcc (per SD)	1.26 (1.01, 1.56)	0.057	1.18 (0.87–1.59)	0.471	1.19 (0.92–1.54)	0.224
ZhangAgeAcc (per SD)	1.27 (0.79, 2.04)	0.341	0.82 (0.34–1.97)	0.771	1.43 (0.78–2.63)	0.263
GrimAgeMortAcc (per SD)	1.69 (1.41, 2.02)	<0.001	1.76 (1.33–2.33)	0.003	1.64 (1.30–2.07)	0.001
GrimAge2MortAcc (per SD)	1.73 (1.43, 2.09)	<0.001	1.72 (1.29–2.31)	0.004	1.75 (1.42–2.17)	<0.001
DunedinPoAm (per SD)	1.26 (1.09, 1.45)	0.005	1.37 (1.14–1.66)	0.007	1.20 (1.01–1.44)	0.083
HorvathTelo (per SD)	0.72 (0.63, 0.81)	<0.001	0.71 (0.47–1.07)	0.228	0.77 (0.64–0.93)	0.023

^1^ Models were adjusted for age, race/ethnicity, smoking history, MetS, and presence of APRI-defined advanced fibrosis. Abbreviation: CI, confidence interval; CLD, chronic liver disease; HR, hazard ratio; SD, standard deviation.

**Table A11:** Associations of DNAm Algorithms with CLD Risk and APRI-Defined Advanced Fibrosis Risk After Additional Adjustment for Total Daily Energy Intake and Healthy Physical Activity.

DNAm Algorithms	Model OR (95% CI)	*p*-Value	FDR q-Value
CLD (1023/2522)			
HorvathAgeAcc (per SD)	1.01 (0.88, 1.17)	0.855	0.994
HannumAgeAcc (per SD)	0.99 (0.86, 1.14)	0.885	0.994
SkinBloodAgeAcc (per SD)	0.95 (0.83, 1.09)	0.457	0.994
PhenoAgeAcc (per SD)	1.00 (0.87, 1.15)	0.994	0.994
LinAgeAcc (per SD)	1.04 (0.89, 1.22)	0.587	0.994
WeidnerAgeAcc (per SD)	1.05 (0.92, 1.20)	0.434	0.994
VidalBraloAgeAcc (per SD)	0.96 (0.77, 1.19)	0.680	0.994
ZhangAgeAcc (per SD)	1.00 (0.62, 1.63)	0.987	0.994
GrimAgeMortAcc (per SD)	0.88 (0.73, 1.07)	0.188	0.751
GrimAge2MortAcc (per SD)	0.88 (0.73, 1.07)	0.185	0.751
DunedinPoAm (per SD)	1.02 (0.89, 1.16)	0.760	0.994
HorvathTelo (per SD)	0.89 (0.76, 1.04)	0.131	0.751
APRI-defined Advanced fibrosis (128/1023)			
HorvathAgeAcc (per SD)	0.96 (0.74, 1.25)	0.759	0.970
HannumAgeAcc (per SD)	1.13 (0.85, 1.48)	0.381	0.916
SkinBloodAgeAcc (per SD)	0.93 (0.73, 1.19)	0.541	0.970
PhenoAgeAcc (per SD)	1.01 (0.74, 1.36)	0.970	0.970
LinAgeAcc (per SD)	0.99 (0.74, 1.33)	0.946	0.970
WeidnerAgeAcc (per SD)	0.98 (0.75, 1.28)	0.872	0.970
VidalBraloAgeAcc (per SD)	0.90 (0.59, 1.35)	0.583	0.970
ZhangAgeAcc (per SD)	1.09 (0.45, 2.63)	0.835	0.970
GrimAgeMortAcc (per SD)	1.27 (0.90, 1.80)	0.161	0.484
GrimAge2MortAcc (per SD)	1.35 (0.98, 1.87)	0.055	0.219
DunedinPoAm (per SD)	1.27 (1.01, 1.58)	0.030	0.178
HorvathTelo (per SD)	0.63 (0.44, 0.90)	0.009	0.106

Models were adjusted for age, sex, race/ethnicity, smoking history, MetS, excess alcohol use, total daily energy intake and healthy physical activity. Abbreviation: CI, confidence interval; CLD, chronic liver disease; DNAm, DNA methylation; OR, odds ratio; SD, standard deviation.

**Table A12:** Cox Regression Survival Models in the Characterized Liver Disease Etiology Subsets After Additional Adjustment for Total Daily Energy Intake and Healthy Physical Activity.

Study Population (Deaths/*n*)	Characterized CLD (403/792)		MetS-Related Liver Disease (237/441)		Alcohol-Associated Liver Disease (145/316)	
	HR (95% CI)	FDR q-Value	HR (95% CI)	FDR q-Value	HR (95% CI)	FDR q-Value
HorvathAgeAcc (per SD)	1.29 (1.13, 1.46)	0.001	1.23 (1.03, 1.46)	0.039	1.35 (1.03, 1.76)	0.055
HannumAgeAcc (per SD)	1.35 (1.19, 1.53)	<0.001	1.33 (1.12, 1.53)	0.005	1.35 (1.12, 1.68)	0.023
SkinBloodAgeAcc (per SD)	1.10 (0.95, 1.28)	0.201	1.09 (0.91, 1.28)	0.394	1.02 (0.91, 1.23)	0.922
PhenoAgeAcc (per SD)	1.36 (1.23, 1.50)	<0.001	1.23 (1.07, 1.50)	0.015	1.61 (1.07, 2.03)	0.001
LinAgeAcc (per SD)	1.14 (0.98, 1.31)	0.118	1.04 (0.86, 1.31)	0.731	1.17 (0.86, 1.37)	0.087
WeidnerAgeAcc (per SD)	0.99 (0.85, 1.15)	0.887	1.03 (0.83, 1.15)	0.805	0.99 (0.83, 1.28)	0.955
VidalBraloAgeAcc (per SD)	1.17 (0.97, 1.43)	0.125	1.20 (0.96, 1.43)	0.146	1.21 (0.96, 1.66)	0.289
ZhangAgeAcc (per SD)	1.61 (0.92, 2.81)	0.119	1.66 (0.90, 2.81)	0.146	1.36 (0.90, 4.18)	0.699
GrimAgeMortAcc (per SD)	1.72 (1.45, 2.04)	<0.001	1.72 (1.31, 2.04)	0.002	1.86 (1.31, 2.45)	0.001
GrimAge2MortAcc (per SD)	1.76 (1.49, 2.09)	<0.001	1.88 (1.47, 2.09)	<0.001	1.83 (1.47, 2.45)	0.001
DunedinPoAm (per SD)	1.31 (1.14, 1.49)	0.001	1.28 (1.06, 1.49)	0.027	1.40 (1.06, 1.65)	0.001
HorvathTelo (per SD)	0.72 (0.62, 0.83)	<0.001	0.73 (0.62, 0.83)	0.005	0.66 (0.62, 0.93)	0.036

Models were adjusted for age, sex, race/ethnicity, smoking history, MetS, presence of APRI-defined advanced fibrosis, total daily energy intake and healthy physical activity. Abbreviation: CI, confidence interval; CLD, chronic liver disease; MetS, metabolic dysfunction; HR, hazard ratio; SD, standard deviation.
